# Th1 responses in vivo require cell-specific provision of OX40L dictated by environmental cues

**DOI:** 10.1038/s41467-020-17293-3

**Published:** 2020-07-09

**Authors:** Dominika W. Gajdasik, Fabrina Gaspal, Emily E. Halford, Remi Fiancette, Emma E. Dutton, Claire Willis, Timo Rückert, Chiara Romagnani, Audrey Gerard, Sarah L. Bevington, Andrew S. MacDonald, Marina Botto, Timothy Vyse, David R. Withers

**Affiliations:** 1 Institute of Immunology and Immunotherapy, College of Medical and Dental Sciences, University of Birmingham, Birmingham, UK; 2 Med. Klinik m.S. Gastroenterologie, Infektiologie und Rheumatologie and Deutsches Rheuma-Forschungszentrum, Charité - Universitätsmedizin Berlin, Berlin, Germany; 3 The Kennedy Institute of Rheumatology, The University of Oxford, Oxford, UK; 4 Institute of Cancer and Genomic Sciences, College of Medical and Dental Sciences, University of Birmingham, Birmingham, UK; 5 Manchester Collaborative Centre for Inflammation Research, Faculty of Biology, Medicine and Health, University of Manchester, Manchester, UK; 6 Department of Medicine, Centre for Inflammatory Disease, Imperial College London, London, UK; 7 Division of Medical and Molecular Genetics and Immunology, Infection and Inflammatory Disease, King’s College London, London, UK

**Keywords:** Immunology, Microbiology

## Abstract

The OX40-OX40L pathway provides crucial co-stimulatory signals for CD4 T cell responses, however the precise cellular interactions critical for OX40L provision in vivo and when these occur, remains unclear. Here, we demonstrate that provision of OX40L by dendritic cells (DCs), but not T cells, B cells nor group 3 innate lymphoid cells (ILC3s), is critical specifically for the effector Th1 response to an acute systemic infection with Listeria monocytogenes (Lm). OX40L expression by DCs is regulated by cross-talk with NK cells, with IFNγ signalling to the DC to enhance OX40L in a mechanism conserved in both mouse and human DCs. Strikingly, DC expression of OX40L is redundant in a chronic intestinal Th1 response and expression by ILC3s is necessary. Collectively these data reveal tissue specific compartmentalisation of the cellular provision of OX40L and define a mechanism controlling DC expression of OX40L in vivo.

## Abstract (web summary)

The OX40-OX40L axis is a crucial component of the costimulatory requirement of CD4 T cell responses. Here, the authors show context and cell type specific expression of OX40L for driving Th1 cell generation during acute and chronic models of infection.

## Introduction

Productive T cell responses require interactions between costimulatory molecules in addition to signals conveyed through T cell receptor (TCR) engagement of peptide:major histocompatibility complex (MHC) complexes. Members of the tumour necrosis factor (TNF) receptor super family (TNFRSF) play critical roles in shaping T cell responses through sustaining proliferation, enhancing survival and directing cellular differentiation^1–5^. CD4 T cell responses are particularly reliant on OX40^6^, with OX40^−/−^ and OX40L^−/−^ mice, alongside OX40^−/−^ TCR transgenic T cells, all implicating OX40 signals in effector memory responses^7–9^. Furthermore, a human patient lacking functional OX40 expression was unable to make rapid effector cytokines upon challenge^10^, entirely consistent with the CD4 T cell defects observed in OX40^−/−^ mice. Thus OX40 signals are thought to be critical in both generating effector CD4 T cell responses and the subsequent establishment of CD4 T cell memory. Since OX40 shares signalling pathways with its fellow TNFRSF member CD30, which is also induced upon T cell activation, there is also potential redundancy in the roles of these two molecules^2,11–13^.

Despite some understanding of the importance of OX40:OX40L at the molecular level, the cellular interactions underpinning provision of this costimulatory signal remain unclear. T helper type 1 (Th1) and Th2 responses are both thought to be OX40 dependent^14–18^, while the generation of Th17 cells may be inhibited by OX40 signals^19^. Expression of OX40L has been described on different professional antigen (Ag)-presenting cells^20,21^ alongside T cells^22^, innate lymphoid cells^23^ and also non-haematopoietic populations^24,25^; thus many different cellular interactions may underpin the phenotypes observed in OX40- and OX40L-deficient mice.

The activation of naive T cells within secondary lymphoid tissue is initiated through T cell interactions with Ag-bearing dendritic cells (DC) within the T zone^26–28^. Thus DCs are obvious potential providers of costimulatory signals to activated T cells. However, after initial interactions with DCs, activated CD4 T cells migrate to the border between B and T zones and the interfollicular spaces, suggesting that some costimulatory ligands maybe provided by further cellular interactions that occur in distinct tissue microenvironments^29^. Cells isolated from secondary lymphoid tissue with the highest expression of OX40L were found to be lymphoid tissue-inducer cells (now considered a type of group 3 innate lymphoid cell (ILC3))^23^ and were located specifically within the interfollicular spaces of lymph nodes (LNs) and the functionally analogous bridging channels of the spleen^30,31^. Given that these ILC3s were also found to express high levels of CD30L^23,32^, they might be key providers of costimulatory signals to activated T cells moving through the interfollicular spaces. Such interactions would be consistent with evidence that ILC3s support splenic CD4 T cell responses^33^. Alternatively or in addition, activated T cells may encounter OX40L expression by B cells^14^ or other T cells^22^. The question of whether specific cellular interactions are required after initial priming by DC remains an important mechanistic detail and answering this would facilitate efforts to manipulate CD4 T cell responses in vivo.

Here we use the reporting of effector cytokines by endogenous Ag-specific CD4 T cells to carefully dissect the provision of OX40L in vivo. Our data reveal that OX40:OX40L interactions are critical for the generation of functional Th1 effector cells in the response to acute systemic *Listeria monocytogenes* (*Lm*) infection, and using conditional OX40L knockout mice, we show that the expression of OX40L by DCs but not by T cells, B cells or ILC3s was required. Expression of OX40L by DCs is dependent upon crosstalk with natural killer (NK) cells that results in early interferon-γ (IFNγ) production directly signalling to the DCs to upregulate OX40L expression. In contrast, within a chronic intestinal Th1 response, DC OX40L expression is redundant and ILC3 expression of OX40L is required. Together, these studies define distinct cellular providers of OX40L in vivo, revealing that compartmentalisation of these signals, dependent on the nature of the response and the microenvironments, occurs to elicit optimal CD4 effector T cell responses.

## Results

### IFNγ-producing Th1 effector T cells are OX40 dependent

**Fig. 1:**
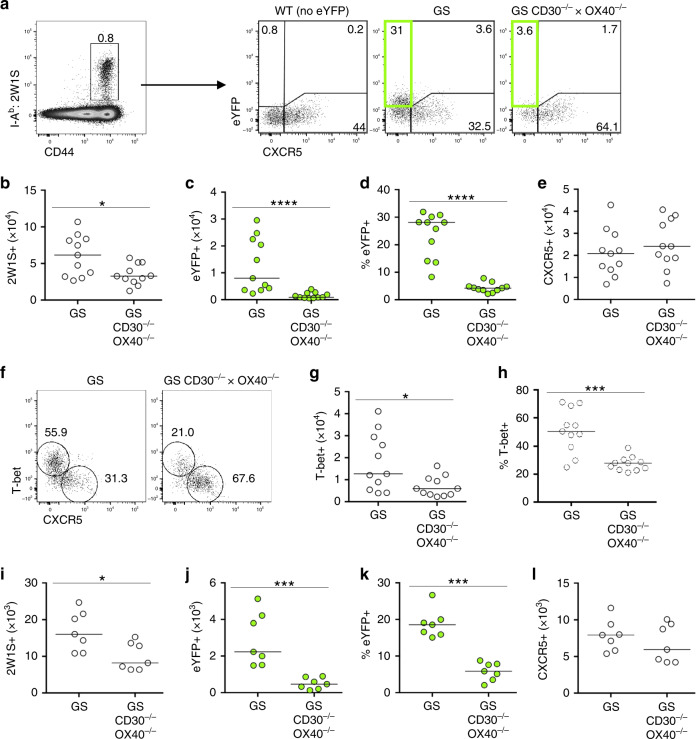
Mice deficient in CD30 and OX40 show a dramatic decrease in reporting of IFNγ production. To determine the requirement for CD30 and OX40 on CD4 T cell effector function without ex vivo manipulation, dual IFNγ (eYFP) and IL-17A reporter mice (Great × Smart17A, GS) sufficient or deficient in CD30 and OX40 (GS CD30^−/−^ × OX40^−/−^) were infected i.v. with *Lm*-2W1S and the splenic response analysed at 7 (**a**–**h**) and 4 (**i**–**l**) dpi. **a** Gating strategy showing identification of 2W1S-specific CD44^hi^ CD4 T cells and their expression of CXCR5 and eYFP in GS and GS CD30^−/−^ × OX40^−/−^ mice. Gated on CD3^+^CD4^+^B220^−^CD11b^−^CD11c^−^ cells; WT cells (lacking any eYFP) used to establish eYFP gating. Data are representative of 11 mice. **b** Enumeration of 2W1S-specific CD4 T cells. **c** Enumeration of eYFP^+^ 2W1S-specific CD4 T cells. **d** Percentage of 2W1S-specific CD4 T cells expressing eYFP. **e** Enumeration of CXCR5^+^ 2W1S-specific CD4 T cells. **f** Expression of T-bet versus CXCR5 by 2W1S-specific CD4 T cells. **g** Enumeration of T-bet^+^ 2W1S-specific CD4 T cells. **h** Percentage of 2W1S-specific CD4 T cells expressing T-bet. Data were pooled from 3 independent experiments (*n* = 11 mice per group). **i** Enumeration of 2W1S-specific CD4 T cells. **j** Enumeration of eYFP^+^ 2W1S-specific CD4 T cells. **k** Percentage of 2W1S-specific CD4 T cells expressing eYFP. **l** Enumeration of CXCR5^+^ 2W1S-specific CD4 T cells. Data were pooled from 2 independent experiments (*n* = 7 mice per group). Values on flow cytometric plots represent percentages; bars on scatter plots represents the median. Statistical significance was tested by using an unpaired, non-parametric, Mann–Whitney two-tailed *T* test: *p ≤ 0.05, ***p ≤ 0.001, ****p ≤ 0.0001.

Studies in humans^10^ and mice^8,34,35^ indicate that effector CD4 T cell responses are highly OX40 dependent. To better understand the costimulatory requirements for CD4 effector cell function in vivo, mice deficient in both CD30 and OX40 (CD30^−/−^ × OX40^−/−^) were crossed with Great × Smart17A dual IFNγ and interleukin (IL)-17A reporter (GS) mice^36^. This approach enables reporting of cytokine expression without ex vivo manipulation. Furthermore, when combined with assessment of endogenous CD4 T cell responses using MHCII tetramers allows for the careful analysis of CD4 T cell function versus impacts on Ag-specific CD4 T cell numbers. Infection with an attenuated *Lm* expressing the 2W1S peptide (*Lm*-2W1S) strain was used to model an acute intracellular bacterial infection that could be tracked with 2W1S-specific MHCII tetramers^37,38^. This well-characterised model provides a robust means to carefully dissect a robust Th1 response in vivo. To assess the impact of CD30 and OX40 deficiency on the effector CD4 T cell response, GS and GS CD30^−/−^ × OX40^−/−^ mice were assessed at 7 days post infection (dpi) with *Lm*-2W1S, revealing a substantial CD44^hi^ 2W1S-specifc CD4 T cell population in both mouse strains, with the absence of CD30 and OX40 resulting in an approximate 2-fold reduction in total Ag-specific CD4 T cells (Fig. 1a, b). Expression of IFNγ, revealed by enhanced yellow fluorescent protein (eYFP) expression, was restricted to CXCR5^−^ 2W1S-specific T cells^38^, and notably, eYFP expression was almost completely absent in CXCR5^−^ 2W1S-specific T cells isolated from GS CD30^−/−^ × OX40^−/−^ mice (Fig. 1c, d). In contrast, the number of CXCR5^+^ follicular 2W1S-specific cells was not significantly changed (Fig. 1e) consistent with previous data that the follicular T cell response to this attenuated *Lm* strain was not OX40 dependent^34^. Expression of the Th1 associated transcription factor T-bet was used to identify effector cells that might be unable to produce effector cytokines (Fig. 1f). Numbers of T-bet^+^CXCR5^−^ 2W1S-specific T cells were reduced approximately 2–3-fold in the absence of CD30 and OX40 (Fig. 1g, h). This is compared to the tenfold loss in eYFP^+^CXCR5^−^ 2W1S-specific T cells indicating that the most substantial defect in the response is the ability of the cells to make effector cytokines. To determine whether the lack of IFNγ-producing Th1 cells at 7 dpi (the peak of the response) reflected an inability of these cells to survive after initial priming, the response was assessed at 4 dpi. Again, eYFP^+^ 2W1S-specific CD4 T cells, but not CXCR5^+^ 2W1S-specific T cells, were heavily reduced in the absence of CD30 and OX40 (Fig. 1i–l) as observed at the peak of the response, suggesting that the effector Th1 cells were not being generated. Combined, these data reveal that, in the primary CD4 T cell response to *Lm*-2W1S infection, the number of Th1 effector CD4 T cells was reduced in the absence of CD30 and OX40, reflecting the early loss of cytokine-producing Th1 effector cells.

**Fig. 2:**
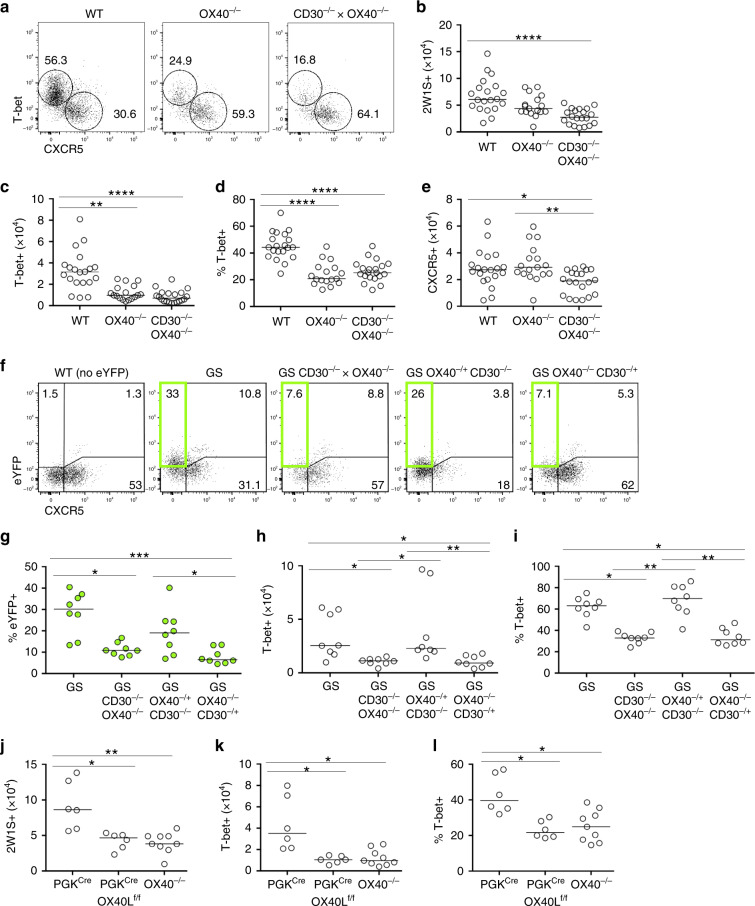
Th1 effector T cells but not CXCR5^+^ populations require OX40:OX40L interactions. The specific contribution of OX40:OX40L interactions in the generation of Th1 effector CD4 T cells was determined in the response at 7 days post infection with *Lm*-2W1S. The 2W1S effector T cell response was assessed in OX40^−/−^ versus CD30^−/−^ × OX40^−/−^ mice. **a** Expression of T-bet versus CXCR5 by 2W1S-specific CD44^hi^ CD4 T cells in WT, OX40^−/−^ and CD30^−/−^ × OX40^−/−^ mice. **b** Enumeration of 2W1S-specific CD4 T cells. **c** Enumeration of T-bet^+^ 2W1S-specific CD4 T cells. **d** Percentage of 2W1S-specific CD4 T cells expressing T-bet. **e** Enumeration of CXCR5^+^ 2W1S-specific CD4 T cells. To dissect the individual requirement for CD30 versus OX40 in the production of IFNγ by effector CD4 T cells, Great × Smart17A × CD30^−/−^ × OX40^−/−^ (GS CD30^−/−^ × OX40^−/−^) mice were crossed with CD30^−/−^ or OX40^−/−^ mice to generate Great × Smart17A mice with only a single functional copy of OX40 (GS CD30^−/−^ × OX40^+/−^) or CD30 (GS CD30^+/−^ × OX40^−/−^). The 2W1S-specific CD4 T cell response at 7 days post infection of these mice alongside controls was then assessed. **f** Gating strategy showing identification of 2W1S-specific CD44^hi^ CD4 T cells and their expression of CXCR5 and eYFP; WT (lacking any eYFP) used to establish eYFP gating. **g** Percentage of 2W1S-specific CD4 T cells expressing eYFP. **h** Enumeration of T-bet^+^ 2W1S-specific CD4 T cells. **i** Percentage of 2W1S-specific CD4 T cells expressing T-bet. Data were pooled from 3 independent experiments (*n* = 8 mice per group). To confirm that mice deficient in OX40L phenocopied mice deficient in OX40, expression of T-bet versus CXCR5 by 2W1S-specific CD44^hi^ CD4 T cells in PGK^cre^, PGK^cre^ × OX40L^f/f^ versus OX40^−/−^ mice was assessed. **j** Enumeration of 2W1S-specific CD4 T cells. **k** Enumeration of T-bet^+^ 2W1S-specific CD4 T cells. **l** Percentage of 2W1S-specific CD4 T cells expressing T-bet. Data were pooled from 2 independent experiments (*n* = 6 PGK^Cre^ mice, *n* = 6 PGK^Cre^ × OX40L^f/f^ mice, *n* = 9 OX40^−/−^ mice). Values on flow cytometric plots represent percentages; bars on scatter plots represents the median. Statistical significance was tested by using an unpaired, non-parametric, Kruskal–Wallis one-way ANOVA with post hoc Dunn’s test: *p ≤ 0.05, **p ≤ 0.01, ***p ≤ 0.001, ****p ≤ 0.0001.

To determine the individual contributions of CD30 and OX40 to the effector T cell response, the 2W1S response at 7 dpi in OX40^−/−^ and CD30^−/−^ × OX40^−/−^ mice was compared (Fig. 2a). Enumeration of total 2W1S-specific CD4 T cells (Fig. 2b), 2W1S-specific Th1 effectors (Fig. 2c, d) and 2W1S-specific CXCR5^+^ follicular cells (Fig. 2e) revealed that loss of OX40 signals accounted for the defects observed in CD30^−/−^ × OX40^−/−^ mice. Thus, for numbers of Ag-specific T cells at the peak of the response, OX40 expression was key. To determine whether CD30 played a role in the functional capacity of the Th1 effector population, GS mice with only single copies of either CD30 or OX40 (GS OX40^−/−^ CD30^−/+^ and GS OX40^−/+^ CD30^−/−^) were also generated. A single copy of OX40 in the absence of CD30 was sufficient for normal IFNγ (eYFP) production (Fig. 2f, g) and formation of a T-bet^+^ effector CD4 T cell population (Fig. 2h, i), indicating that these aspects of the response were not CD30 dependent. Combined, these experiments demonstrate that signals through OX40, rather than through CD30, were critical for the primary Th1 response induced by *Lm*-2W1S infection. While OX40L is the only reported ligand for OX40, we confirmed that observations made in OX40^−/−^ mice were consistent with mice lacking OX40L by crossing OX40L^f/f^ mice^39^ with PGK^cre^ mice (which express cre recombinase ubiquitously) to generate a total OX40L-deficient mouse model. As anticipated, total OX40^−/−^ or PGK^cre^ × OX40L^f/f^ mice showed a comparable disruption of the effector Th1 response (Fig. 2j–l).

### DC expression of OX40L is required in systemic Th1 response

**Fig. 3:**
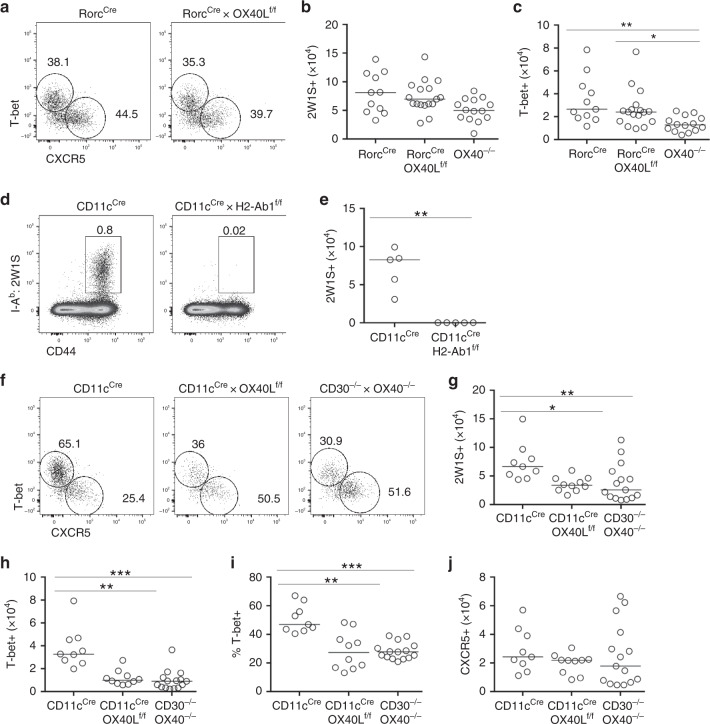
Expression of OX40L by DC is required for effector Th1 responses. To investigate the critical cellular interaction for generation of Th1 effector T cells, conditional OX40L-deficient mice targeting ILC3s (Rorc^cre^) or DCs (CD11c^cre^) were generated. **a** Expression of T-bet versus CXCR5 by 2W1S-specific CD44^hi^ CD4 T cells in Rorc^cre^ and Rorc^cre^ × OX40L^f/f^ mice. **b** Enumeration of CD44^hi^ 2W1S-specific CD4 T cells in Rorc^cre^, Rorc^cre^ × OX40L^f/f^ and OX40^−/−^ control mice. **c** Enumeration of T-bet^+^ 2W1S-specific CD44^hi^ CD4 T cells in Rorc^cre^, Rorc^cre^ × OX40L^f/f^ and OX40^−/−^ control mice. Data were pooled from 3 independent experiments (*n* = 11 Rorc^cre^ mice, *n* = 17 Rorc^cre^ × OX40L^f/f^ mice, *n* = 14 OX40^−/−^ mice). **d** Representative flow cytometric plots showing CD44^hi^ 2W1S-specific CD4 T cells in the spleen of CD11c^cre^ and CD11c^cre^ × H2-Ab1^f/f^ mice at 7 days post infection. **e** Enumeration of CD44^hi^ 2W1S-specific CD4 T cells. Data are representative of 2 independent experiments, *n* = 5 mice per group. **f** Expression of T-bet versus CXCR5 by 2W1S-specific CD44^hi^ CD4 T cells in CD11c^cre^, CD11c^cre^ × OX40L^f/f^ and CD30^−/−^ × OX40^−/−^ mice. **g** Enumeration of 2W1S-specific CD44^hi^ CD4 T cells. **h** Enumeration of T-bet^+^ 2W1S-specific CD44^hi^ CD4 T cells. **i** Percentage of 2W1S-specific CD4 T cells expressing T-bet. **j** Enumeration of CXCR5^+^ 2W1S-specific CD44^hi^ CD4 T cells. Data were pooled from 3 independent experiments (*n* = 9 CD11c^cre^ mice, *n* = 10 CD11c^cre^ × OX40L^f/f^ mice, *n* = 15 CD30^−/−^ × OX40^−/−^ mice). Values on flow cytometric plots represent percentages; bars on scatter plots represents the median. Statistical significance was tested in **e** by using an unpaired, non-parametric, Mann–Whitney two-tailed *T* test and in **b**, **c**, **h**–**j** by using Kruskal–Wallis one-way ANOVA with post hoc Dunn’s test: *p ≤ 0.05, **p ≤ 0.01, ***p ≤ 0.001.

The specific cellular providers of OX40L signals within secondary lymphoid tissue remain to be elucidated, with professional Ag-presenting cells, T cells and ILC populations all possible sources in vivo. We hypothesised that in the response to *Lm*-2W1S there was a critical cellular provider of OX40L signals that underpinned the CD4 effector T cell response. To test this, OX40L^f/f^ mice were crossed with multiple cell-specific cre strains to target DCs, ILC3s, T cells and B cells, since all of these have been reported to express OX40L^5^. Specific deletion of OX40L expression on the appropriate populations was confirmed through analysis of splenocytes ex vivo alongside fate-mapping of cre expression (Supplementary Fig. 1). Given their expression of OX40L^23,32^, positioning in lymphoid tissue^31^ and support for splenic CD4 T cell responses^33^, we first hypothesised that ILC3 expression of OX40L was required for functional Th1 responses. To test this, Rorc^cre^ versus Rorc^cre^ × OX40L^f/f^ mice were infected with *Lm*-2W1S and assessed at 7 dpi, alongside OX40^−/−^ controls. Strikingly, no defect was observed in the 2W1S-specific response when ILC3 lacked OX40L (Fig. 3a–c), indicating that ILC3 provision of OX40L was not required for the generation of effector Th1 responses in the spleen. Given that the defect in the Th1 effector response was established by 4 dpi, we hypothesised that DCs were the critical source of OX40L signal in vivo. To first confirm that the 2W1S-specific response to *Lm*-2W1S was dependent upon interactions with DCs, MHCII expression by DCs was deleted using CD11c^cre^ × H2Ab-1^f/f^ mice. As expected, in the absence of MHCII expression on CD11c^+^ cells, the 2W1S-specific response was completely abrogated (Fig. 3d, e). To then test whether DC provision of OX40L was necessary for normal Th1 effector T cell responses, CD11c^cre^ × OX40L^f/f^ mice were infected with *Lm*-2W1S, alongside CD11c^cre^ and CD30^−/−^ × OX40^−/−^ controls. Compared with CD11c^cre^ controls, CD11c^cre^ × OX40L^f/f^ mice had significantly reduced numbers of 2W1S-specific CD4 T cells at 7 dpi (Fig. 3f, g) due to substantial loss of the T-bet^+^CXCR5^−^ subset (Fig. 3h–j). Strikingly, the defects in CD11c^cre^ × OX40L^f/f^ mice were comparable to those observed in CD30^−/−^ × OX40^−/−^ controls, demonstrating that the expression of OX40L by DCs was absolutely required for the Th1 effector response and could account for the impaired Th1 effector response observed in the total absence of CD30 and OX40 signals. While these data clearly establish the importance of OX40L expression by DCs, we sought to further confirm that there was no absolute requirement for provision of OX40L by lymphocytes during the primary response, perhaps acting subsequent to interactions with DCs. Therefore, B cell-specific (Mb1^cre^) and T cell-specific (CD4^cre^) conditional OX40L-deficient mice were infected with *Lm*-2W1S and the 2W1S-specific CD4 T cell response was analysed. No significant differences were observed versus cre-only controls in either strain confirming that there was no requirement for B nor T cell provision of OX40L in vivo for the normal response to *Lm*-2W1S (Supplementary Fig. 2). Together, these data reveal that, in the primary response to *Lm*-2W1S, provision of OX40L by a single cellular source, the CD11c^+^ DCs, is required for the Th1 effector T cell response.

### Early IFNγ regulates OX40L expression by DCs

**Fig. 4:**
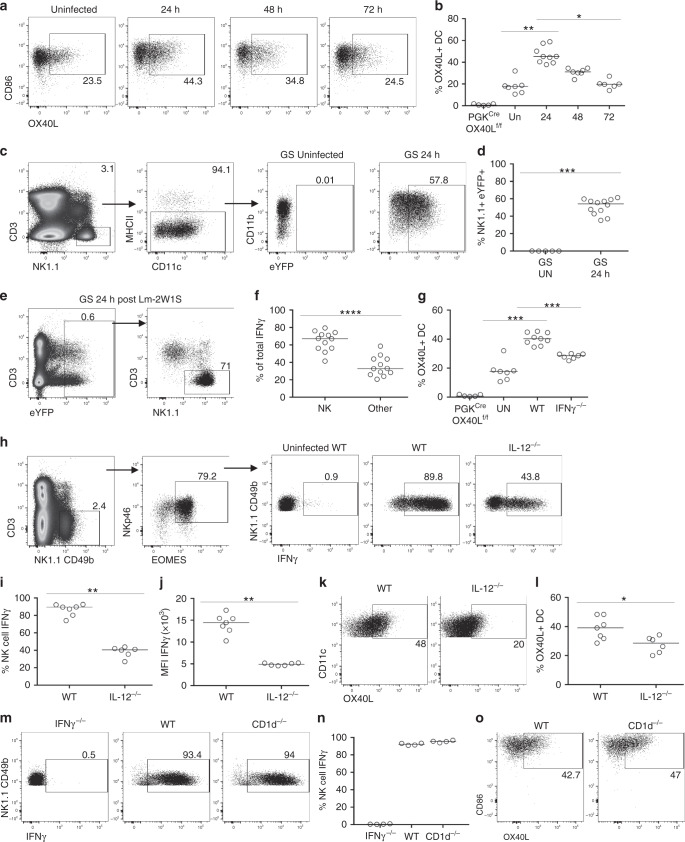
OX40L is upregulated on DCs in response to IL-12-mediated production of IFNγ by NK cells. Splenocytes were isolated from mice at different times post infection with *Lm*-2W1S. **a** Expression of OX40L by DC (lin^−^(CD3, B220, CD49b), CD11c^+^, MHCII^+^, CD86^+^, SIRPα^+^) from uninfected mice and mice infected with *Lm*-2W1S 24, 48 and 72 h previously. **b** Proportion of DC-expressing OX40L at different times post infection with *Lm*-2W1S, versus uninfected and PGK^cre^ × OX40L^f/f^ controls. Data are representative of 2 independent experiments (*n* = 5 PGK^cre^ × OX40L^f/f^ mice, *n* = 7 UN WT mice, *n* = 9 WT mice at 24 h post *Lm*-2W1S, *n* = 7 WT mice at 48 h post *Lm*-2W1S, *n* = 6 WT mice at 72 h post *Lm*-2W1S infection). Great × Smart17A mice were used to assess IFNγ production at 24 h post infection. **c** Representative flow cytometric plots showing the expression of IFNγ by NK cells. **d** Percentage of NK cells expressing IFNγ (eYFP). **e** Representative flow cytometric plots showing the expression of IFNγ by different immune cells. **f** Percentage of total IFNγ attributable to NK cells or other immune cell populations. Data were pooled from 2 independent experiments (*n* = 5 GS UN mice, *n* = 12 GS mice at 24 post *Lm*-2W1S). **g** Splenocytes from WT and IFNγ^−/−^ mice were assessed for OX40L expression at 24 h post infection with *Lm*-2W1S, alongside PGK^cre^ × OX40L^f/f^ and uninfected (UN) controls. Data were pooled from 2 independent experiments (*n* = 5 PGK^cre^ × OX40L^f/f^ mice, *n* = 7 UN WT mice, *n* = 9 WT mice, *n* = 7 IFNγ^−/−^ mice). **h** Representative flow cytometric plots showing production of IFNγ by NK cells from uninfected WT and infected WT and IL-12p35^−/−^ mice 24 h post *Lm*-2W1S. **i**, **j** Proportion of NK cells producing IFNγ and MFI of IFNγ 24 h post infection with *Lm*-2W1S. **k** Representative flow cytometric plots showing OX40L expression on DC in WT and IL-12p35^−/−^ mice 24 h post infection with *Lm*-2W1S. **l** Percentage of DC-expressing OX40L in WT and IL-12p35^−/−^ mice 24 h post infection with *Lm*-2W1S. Data are representative of 1 experiment (*n* = 7 WT mice, *n* = 6 IL-12p35^−/−^ mice). **m** Representative flow cytometric plots showing the expression of IFNγ by splenic NK cells from IFNγ^−/−^, WT and CD1d^−/−^ mice 24 h after infection with *Lm*-2W1S. **n** Percentage of NK cells expressing IFNγ. **o** Expression of OX40L by DC from WT and CD1d^−/−^ mice 24 h after infection with *Lm*-2W1S. Data are representative of 1 experiment (*n* = 4 mice per group). Values on flow cytometric plots represent percentages; bars on scatter plots represents the median. Statistical significance was tested in **b**, **g** by using Kruskal–Wallis one-way ANOVA with post hoc Dunn’s test and in **d**, **i**, **j**, **l** by using an unpaired, non-parametric, Mann–Whitney two tailed *T* test: *p ≤ 0.05, **p ≤ 0.01, ***p ≤ 0.001, ****p ≤ 0.0001.

Defining the cellular interaction required for provision of OX40L enabled the investigation into how expression of this key costimulatory pathway was regulated in vivo. The kinetics of DC expression of OX40L was assessed by infecting mice with *Lm*-2W1S and then analysing splenic DC expression of OX40L at different time points post infection. Although a basal level of OX40L expression by the splenic DCs was evident, *Lm*-2W1S infection resulted in the rapid upregulation of OX40L expression by 24 h, which then declined to basal levels once more by 72 h post infection (Fig. 4a, b). The dynamics of OX40 expression relative to OX40L expression in the same spleen tissue was then assessed to better understand the relationship between maximal DC expression of the ligand and Ag-specific T cell expression of the receptor (Supplementary Fig. 3). OX40 expression was evident on 2W1S-specific CD4 T cells at 48 h, where approximately 50% of the cells expressed OX40 and many also co-expressed CD25, described to be expressed by early Th1 effector T cells^38^. The proportion of OX40-expressing 2W1S-specific CD4 T cells peaked at 2 dpi and then rapidly decline such that by 4 dpi the vast majority of the responding CD4 T cells lacked any detectable expression of OX40 and DC expression of OX40L had returned to baseline. Thus the expression of both OX40 and OX40L expression was focused very early in the response and OX40 expression was limited to only a proportion of the responding 2W1S-specific CD4 T cells with a phenotype consistent with Th1 effector cells.

While OX40L expression can be upregulated in vitro by stimulation through CD40^21^, the rapid upregulation of OX40L expression observed after *Lm*-2W1S infection suggested that early signals from innate immune cells would precede CD40L expression by activated T cells. Activation of NK cells contributes to robust Th1 responses^40–42^, and more recently, IFNγ release by activated NK cells was revealed as the mechanism by which an adjuvanted vaccine response enhanced Th1 responses^43^. Furthermore, crosstalk between NK cells and DCs has been previously identified as a key mechanism through which both populations are fully activated^44^. To investigate early innate cell production of IFNγ in vivo, GS mice were infected with *Lm*-2W1S and assessed 24 h later. Gating on NK cells revealed substantial IFNγ expression compared to NK cells from uninfected GS control mice (Fig. 4c, d). Moreover, NK cells were the dominant source (approximately 70%) of IFNγ expression at this time, with T cells contributing most of the remaining signal (Fig. 4e, f). To ask whether this early IFNγ signal was required for upregulation of OX40L on DCs, wild-type (WT) and IFNγ^−/−^ mice were infected with *Lm*-2W1S and DC expression of OX40L assessed 24 h later. Notably, the upregulation of OX40L expression by DCs isolated from IFNγ^−/−^ mice was impeded compared to WT controls but was higher than the basal level observed in uninfected mice (Fig. 4g). These data indicate that early IFNγ was resulting in the increase in OX40L expression by DCs, but other signals might also contribute. In vitro, Th1 responses to heat-killed *Lm* required IL-12^45,46^, and neutralisation of IL-12 in vivo results in reduced resistance to *Lm*, which could be reversed by recombinant IFNγ^47^. To assess the requirement for IL-12, IL-12p35^−/−^ mice were infected with *Lm*-2W1S and assessed 24 h later for NK cell production of IFNγ and OX40L expression by splenic DCs. The proportion of NK cells producing IFNγ (Fig. 4h, i) and the amount of IFNγ produced (Fig. 4j) were significantly reduced in IL-12p35^−/−^ mice and DC upregulation of OX40L was impaired (Fig. 4k, l), indicating that IL-12-mediated NK cell expression of IFNγ was linked to DC OX40L expression. We further confirmed that expression of IL-12 mRNA was equally upregulated by DCs from CD11c^cre^ × OX40L^f/f^ and OX40L^f/f^ littermate controls after infection with *Lm*-2W1S (Supplementary Fig. 4), indicating that IL-12 production by OX40L-deficient DCs was not impaired and the loss of Th1 effector cells not due to impaired polarisation during priming.

Since the activation of NK cells can occur subsequent to the rapid activation of invariant NK T (iNKT) cells and mediated through very early iNKT expression of IFNγ^48^, we assessed the requirement for iNKT cell activation using CD1d^−/−^ mice. WT, CD1d^−/−^ and IFNγ^−/−^ mice were infected with *Lm*-2W1S, and NK cell IFNγ expression was assessed 24 h later. NK cells from CD1d^−/−^ mice showed comparable IFNγ expression to NK cells from WT controls (Fig. 4m, n). Furthermore, DC expression of OX40L was also not impaired in CD1d^−/−^ mice (Fig. 4o). Combined, these data reveal that iNKT cells are not required for IFNγ production by NK cells in response to *Lm*-2W1S and highlight the importance of IFNγ in enhancing DC expression of OX40L.

### IFNγ produced by NK cells signals directly to DCs

**Fig. 5:**
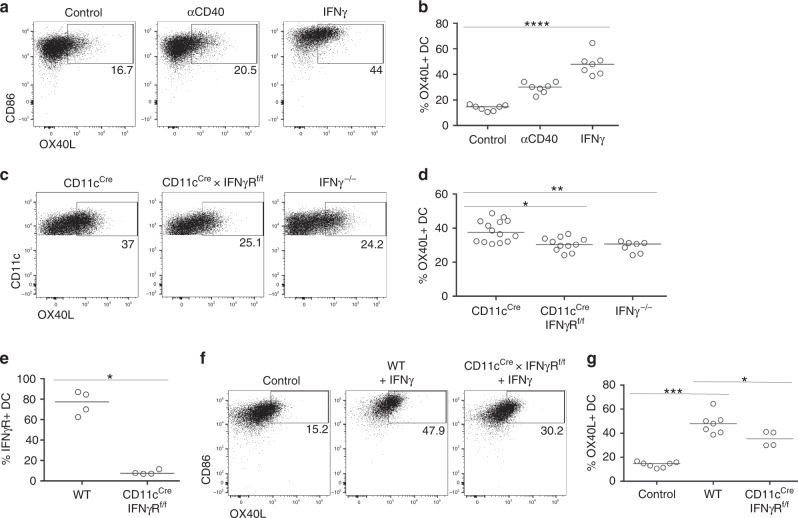
IFNγ directly enhances OX40L expression on DCs. To determine the mechanism of how IFNγ caused the upregulation of OX40L on DCs, in vitro and in vivo experiments to test a direct interaction were performed. **a** Representative flow cytometric plots showing the expression of OX40L by splenic DCs cultured with either anti-CD40 Abs or recombinant IFNγ. **b** Percentage of DC-expressing OX40L after culture. Data were pooled from 2 independent experiments, *n* = 7. **c** Representative flow cytometric plots showing the expression of OX40L on DCs from CD11c^Cre^, CD11c^Cre^ × IFNγR^f/f^ and IFNγ^−/−^ mice 24 h after infection with *Lm*-2W1S. **d** Proportion of DC-expressing OX40L 24 h post infection with *Lm*-2W1S. Data are representative of 3 independent experiments (*n* = 14 CD11c^Cre^ mice, *n* = 11 CD11c^Cre^ × IFNγR^f/f^ mice, *n* = 7 IFNγ^−/−^ mice). **e** Proportion of DC-expressing IFNγR in WT versus CD11c^Cre^ × IFNγR^f/f^ mice. Data are representative of 2 independent experiments (*n* = 4 mice per group). **f** Representative flow cytometric plots showing the expression of OX40L by DCs isolated from WT versus CD11c^Cre^ × IFNγR^f/f^ mice and cultured with recombinant IFNγ. **g** Proportion of DC-expressing OX40L after culture with recombinant IFNγ. Data are representative of 2 independent experiments (*n* = 7 WT mice, *n* = 4 CD11c^Cre^ × IFNγR^f/f^ mice). Values on flow cytometric plots represent percentages; bars on scatter plots represents the median. Statistical significance was tested in **b**, **d** by using Kruskal–Wallis one-way ANOVA with post hoc Dunn’s test and in **e**, **g** by using an unpaired, non-parametric, Mann–Whitney two-tailed *T* test: *p ≤ 0.05, **p ≤ 0.01, ***p ≤ 0.001, ****p ≤ 0.0001.

To investigate whether IFNγ could directly upregulate OX40L expression by DCs, splenocytes were cultured in vitro with recombinant IFNγ alongside anti-CD40 antibodies (Abs) as a positive control^21^. Culture with IFNγ induced robust upregulation of OX40L expression by DCs (Fig. 5a, b), suggesting that IFNγ might directly stimulate OX40L expression via signals through the IFNγR. To test this in vivo, CD11c^cre^ × IFNγR^f/f^ mice were generated and infected with *Lm*-2W1S alongside CD11c^cre^ and IFNγ^−/−^ controls. DCs isolated from either CD11c^cre^ × IFNγR^f/f^ or total IFNγ^−/−^ mice expressed significantly less OX40L than controls (Fig. 5c, d). Flow cytometric analysis of IFNγR expression by CD11c^+^ DCs confirmed efficient deletion of this receptor in CD11c^cre^ × IFNγR^f/f^ mice (Fig. 5e). Finally, DCs from CD11c^cre^ × IFNγR^f/f^ mice cultured with recombinant IFNγ failed to upregulate OX40L to the level observed for WT controls (Fig. 5f, g). Combined, these data indicate a mechanism where DC-expressed IL-12 instructs NK production of IFNγ, which then directly signals back to the DC to enhance OX40L expression and orchestrate the generation of a robust Th1 effector T cell response.

**Fig. 6:**
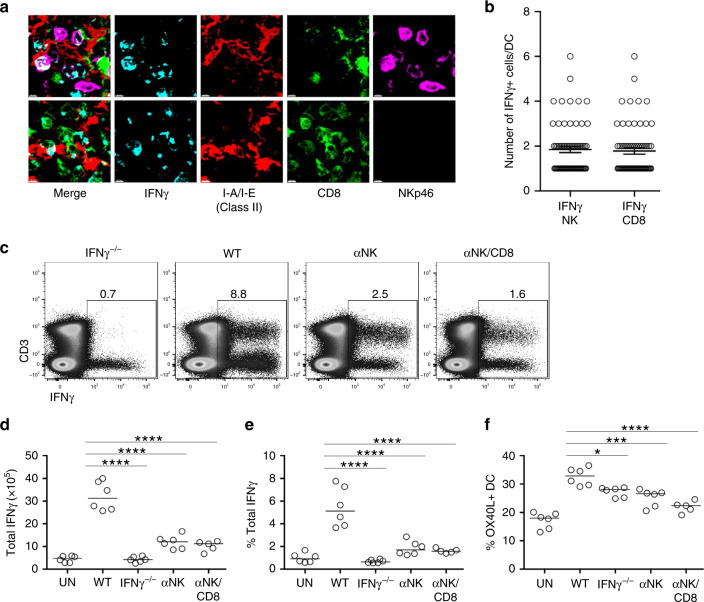
NK cells are a critical source of IFNγ in vivo. To explore the spatial distribution of cells making IFNγ after infection with *Lm*, WT mice were infected with *Lm* and killed 24 h after infection (with treatment with brefeldin A 6 h before spleen harvest). Spleen sections were stained with anti-CD8 (green), Nkp46 (NK, magenta), I-A/I-E (DC, red) and IFNγ (cyan) antibodies. **a** Photographs are representative examples of IFNγ-producing CD8 and NK cells in close association with dendritic cells, scale bar: 4 μm. Data are representative of 2 independent experiments. **b** Quantification of the number of IFNγ-producing CD8 T cells (*n* = 67) and IFNγ-producing NK cells (*n* = 70) in contact per dendritic cell (*n* = 112) in 2 different spleens; mean and sem are shown, data were pooled from 2 independent experiments. To investigate the specific role of early IFNγ production by NK cells or CD8 T cells, WT mice were given αNK1.1 or αNK1.1 and αCD8 monoclonal antibodies prior to the infection with *Lm*-2W1S. The IFNγ responses and OX40L expression on DC were assessed 24 h post infection and were compared to uninfected WT, infected WT and infected IFNγ^−/−^ mice. **c** Representative flow cytometric plots showing total production of IFNγ at 24 h post infection. **d** Total numbers of IFNγ^+^ cells. **e** Percentages of IFNγ^+^ cells. **f** Proportion of dendritic cell-expressing OX40L at 24 h post infection. Data were pooled from 2 independent experiments (*n* = 6 UN WT mice, *n* = 6 WT mice, *n* = 6 IFNγ^−/−^ mice, *n* = 6 αNK WT mice, *n* = 5 αNK/CD8 WT mice). Statistical significance was tested by using ordinary one-way ANOVA with post hoc Tukey’s tests, following successful normality tests: *p ≤ 0.05, ***p ≤ 0.001, ****p ≤ 0.0001.

Although NK cells were the largest population of IFNγ-expressing cells, CD3^+^ IFNγ^+^ cells were evident 24 h after infection with *Lm*-2W1S raising the possibility that multiple sources of IFNγ expression contributed to DC activation. To better understand the spatial distribution of IFNγ-producing cells in the spleen after *Lm* infection, IFNγ expression was localised in tissue sections 24 h post infection. Expression of IFNγ was detected within some white pulp areas of the spleen of infected mice (Supplementary Fig. 5). Both IFNγ^+^ NK cells and IFNγ^+^ CD8 T cells were detected in approximately equal frequency in the proximity of DCs in the splenic white pulp (Fig. 6a, b). To specifically test in vivo whether NK cells were a critical source of IFNγ, mice were treated with either anti-NK1.1 Abs alone or in combination with anti-CD8 Abs, then infected with *Lm*-2W1S and analysed 24 h later. Depletion of the targeted cells and a loss IFNγ expression among this population was confirmed by flow cytometry (Supplementary Fig. 6), and the expected reduction in IFNγ expression was observed when splenocytes were analysed after ex vivo culture in the presence of brefeldin A (Fig. 6c–e). Importantly, depletion of NK cells resulted in the impaired upregulation of OX40L expression by DCs, comparable to that observed in IFNγ^−/−^ controls (Fig. 6f), indicating that NK cells were a critical in vivo source of the cytokine. Given the proximity of IFNγ^+^ CD8 T cells to DCs in the splenic white pulp of *Lm*-2W1S-infected mice, mice were also treated with a combination of anti-NK1.1 and anti-CD8 Abs; however, this did not significantly reduce the OX40L expression by DCs further.

While our data indicated an important role for IFNγ in regulating OX40L expression, the OX40L expression by DC when IFNγ signalling was disrupted remained higher than in uninfected mice, indicating that other signals likely contributed to the upregulation of this ligand. To investigate this further, we employed in vitro cultures of splenocytes to screen other cytokines that could boost the expression of OX40L by DCs. Splenocytes from WT and IFNγ^−/−^ mice were compared in parallel to help determine potentially indirect effects via stimulating IFNγ expression in the cultures. Notably, basal expression of OX40L in IFNγ^−/−^ splenocyte cultures was lower than in WT controls. Recombinant IFNγ robustly upregulated OX40L expression as observed previously, but both IL-18 and TNFα were identified as further cytokines that could enhance OX40L expression by DCs in this assay (Supplementary Fig. 7).

### NK cell-derived IFNγ upregulates OX40L on human DCs

**Fig. 7:**
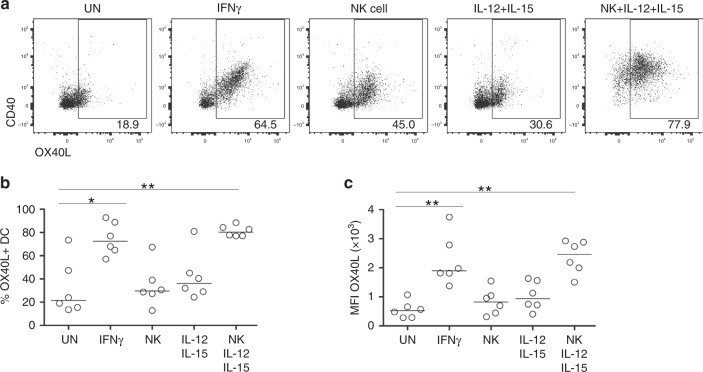
Human cDC2 upregulate OX40L after treatment with recombinant IFNγ or co-culture with autologous NK cells activated by IL-12/15. To investigate whether NK cell crosstalk regulated OX40L expression on human DCs, cDC2 and NK cells were isolated to high purity by FACS and cultured in vitro. **a** Representative flow cytometric plots showing the expression of OX40L and CD40 on cDC2 (CD14^−^ CD19^−^ CD3^−^ HLA-DR^+^ CD1c^+^) after 40 h of culture in the indicated conditions. **b** Percentage of cDC2-expressing OX40L under the conditions indicated. **c** MFI of OX40L expression by cDC2 under the conditions indicated. Data were pooled from 3 independent experiments (*n* = 6 patient samples). Values on flow cytometric plots represent percentages; bars on scatter plots represent the median. Statistical significance was tested by using Kruskal–Wallis one-way ANOVA with post hoc Dunn’s test: *p ≤ 0.05, **p ≤ 0.01.

Having identified how DC expression of OX40L in response to *Lm*-2W1S infection was controlled in vivo, we sought to confirm that this pathway was conserved in human DCs. Human cDC2 were isolated to high purity by fluorescence-activated cell sorting (FACS) and then cultured alone or with autologous FACS-isolated NK cells (Supplementary Fig. 8). Under these conditions, recombinant human IFNγ was sufficient to significantly enhance OX40L expression consistent with direct effects of this cytokine on human cDC2 (Fig. 7a–c). Activation of NK cells in co-culture with cDC2 through addition of IL-12 and IL-15 was also able to enhance OX40L expression to similar levels as the recombinant IFNγ. Thus the mechanism of direct IFNγ signalling to DCs resulting from crosstalk with NK cells would also appear to operate for human DCs.

### DC expression of OX40L redundant in intestinal Th1 response

**Fig. 8:**
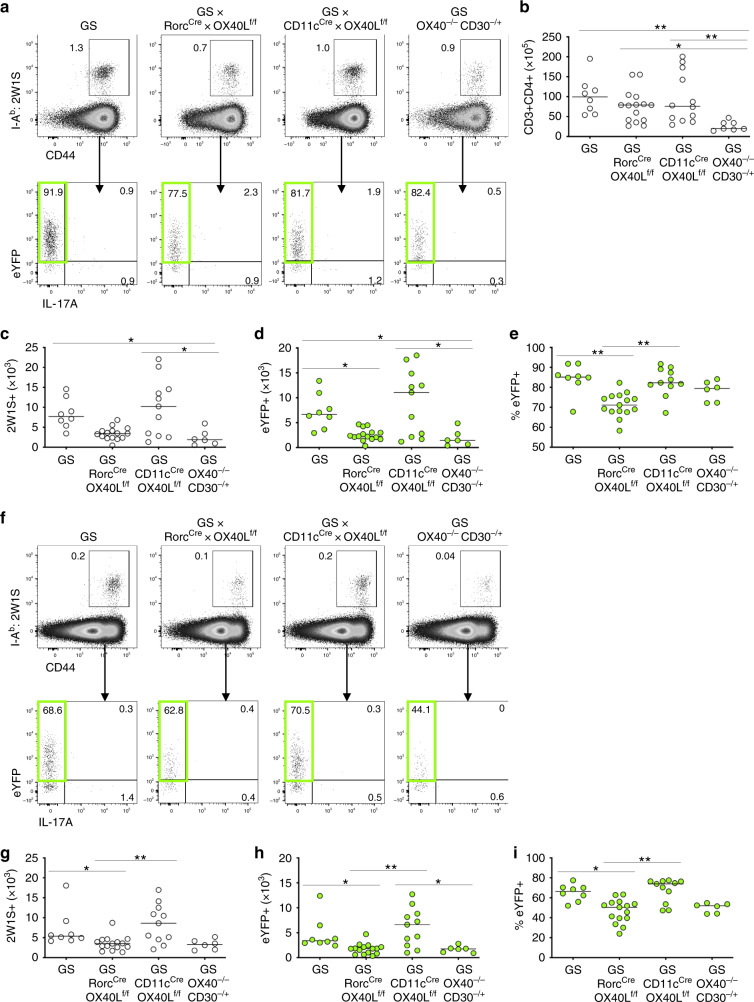
DC provision of OX40L is redundant for efficient effector CD4 T cell responses to *Salmonella typhimurium*-2W1S. To investigate the role of OX40L signals in other Th1 infection models, GS, GS × Rorc^cre^ × OX40L^f/f^, GS × CD11c^cre^ × OX40L^f/f^ and GS × OX40^−/−^ × CD30^−/+^ reporter mice were infected with intestinal pathogen *Salmonella*-2W1S via oral gavage 24 h post administration of streptomycin and the responses in the colon and mLN were assessed 7 days later. **a** Flow cytometric plots showing 2W1S-specific CD44^hi^ CD4 T cell response and the eYFP expression in the colon of GS, GS × Rorc^cre^ × OX40L^f/f^, GS × CD11c^cre^ × OX40L^f/f^ and GS × OX40^−/−^ × CD30^−/+^ mice at D7 post infection. **b** Enumeration of CD4 T cells in the colon. **c** Enumeration of 2W1S-specific CD4 T cells in colon. **d** Enumeration of eYFP^+^ 2W1S-specific CD4 T cells. **e** Percentage of 2W1S-specific CD4 T cells expressing eYFP. **f** Flow cytometric plots showing 2W1S-specific CD44^hi^ CD4 T cell response and the eYFP expression in the mesenteric LN of GS, GS × Rorc^cre^ × OX40L^f/f^, GS × CD11c^cre^ × OX40L^f/f^ and GS × OX40^−/−^ × CD30^−/+^ mice at D7 post infection. **g** Enumeration of 2W1S-specific CD4 T cells in the mesenteric LN. **h** Enumeration of eYFP^+^ 2W1S-specific CD4 T cells. **i** Percentage of 2W1S-specific CD4 T cells expressing eYFP. Data were pooled from 3 independent experiments (*n* = 8 GS mice, *n* = 15 GS × Rorc^cre^ × OX40L^f/f^ mice, *n* = 11 GS × CD11c^cre^ × OX40L^f/f^ mice, *n* = 6 GS × OX40^−/−^ × CD30^−/+^ mice). Values on flow cytometric plots represent percentages; bars on scatter plots represents the median. Statistical significance was tested by using Kruskal–Wallis one-way ANOVA with post hoc Dunn’s test: *p ≤ 0.05, **p ≤ 0.01.

Our data have revealed that OX40L expression by DCs, licenced by early crosstalk with NK cells, is sufficient for Th1 cell expansion and function in a model of acute systemic bacterial infection. To ask whether this mechanism was specific to the splenic response observed with *Lm*-2W1S infection, we sought to test the requirement for DC OX40L under quite different experimental conditions. To this end, we established an oral *Salmonella* infection model using an attenuated *Salmonella*-2W1S strain^49^, thus enabling tracking of the same endogenous 2W1S-specific CD4 T cell response within the draining secondary lymphoid tissue (mesenteric LNs (mLNs)) and the non-lymphoid effector site (colon). Having established that OX40L expression by ILC3s was not required in the response to *Lm*-2W1S, the *Salmonella* infection model provided an opportunity to further test the potential role of ILC3s in supporting CD4 T cells at mucosal sites via their expression of OX40L^50^. Thus CD11c^cre^ × OX40L^f/f^ and Rorc^cre^ × OX40L^f/f^ mice, alongside littermate OX40L^f/f^ only and OX40-deficient controls, were infected, and the 2W1S-specific response was assessed 7 days later in the colon (Fig. 8a). To aid assessment of cytokine production, the different OX40L conditional mice had been further crossed with Great × Smart17A dual IFNγ and IL-17A reporters. Enumeration of the total CD4 T cell response confirmed a significant reduction in the intestinal CD4 T cell compartment in mice lacking OX40 (Fig. 8b)^51^, which was not detected in the conditional OX40L-deficient mice. Strikingly, the number of 2W1S-specific CD4 T cells, alongside the proportion and number of IFNγ^+^ (eYFP^+^), was specifically reduced in Rorc^cre^ × OX40L^f/f^, but not in CD11c^cre^ × OX40L^f/f^ mice (Fig. 8c–e), indicating that, for the effector Th1 cells in the colon, OX40L expression by ILC3s rather than by DCs was required. Furthermore, assessment of the response in the mLN (Fig. 8f) again revealed that the 2W1S Th1 response was specifically reduced in Rorc^cre^ × OX40L^f/f^ mice (Fig. 8g–i), suggesting that ILC3s might provide OX40L within the draining lymphoid tissue, either in addition or instead of provision within the colon. Regardless, the data clearly showed that, in the primary response to *Salmonella*-2W1S, the expression of OX40L by DCs was not required to sustain the effector Th1 response.

Collectively these data demonstrate a critical role for OX40L in supporting the effector Th1 response and that in vivo there are specific cellular providers of these signals dependent on the microenvironment and the response.

## Discussion

Many cells are able to present Ag to T cells and potentially provide the costimulatory signals critical for optimal responses^52^. This suggests that CD4 T cells may interact with distinct Ag-presenting cell populations during the initial stages of the immune response as they move through different microenvironments within secondary lymphoid tissue^29^. However, despite its relevance for understanding how CD4 T cell responses are regulated, such a model has not been tested in vivo. Here we have provided a detailed in vivo dissection of the cellular provision of OX40L for Th1 effector CD4 T cells contrasting an acute systemic response with a chronic intestinal infection. Our data reveal that, in an acute Th1 infection model, OX40L provision by DCs is critical with OX40L expression by T cells, B cells or ILC3s redundant for the Th1 effector T cell response. In contrast, DC OX40L expression was not needed for the Th1 effector cells in response to oral *Salmonella* infection and here OX40L expression by ILC3s, or potentially T cells, was absolutely required. Thus, through conditionally deleting OX40L on different immune cell populations, our data reveal that, in vivo, there are specific cellular interactions that are critical for normal OX40L provision and this is tissue and response specific.

Having identified that DC expression of OX40L was required for generating a robust Th1 response, we were then able to dissect the pathway through which expression of OX40L by the DC was regulated, thereby identifying that NK cell:DC crosstalk ensures optimal OX40L expression through early IFNγ production signalling back to the DC. That this mechanism was also evident for regulating human DC expression of OX40L suggests this is a broadly relevant pathway controlling the expression of this costimulatory molecule. However, it is evident that other pro-inflammatory cytokines can also enhance DC expression of OX40L and in vitro culture with recombinant IL-18 or TNFα enhanced OX40L expression by DCs and both of these cytokines are required for optimal clearance of virulent *Lm* infection^53,54^. That DC expression of OX40L was critical in the response to *Lm*-2W1S likely reflects the very acute nature of this attenuated infection, resulting in only a short window of OX40 expression by activated T cells and thus presumably limited time for different cellular interactions providing OX40L signals. Where high levels of Ag are maintained for longer, such as in more chronic infections, T cell expression of OX40 is prolonged and here other cellular interactions after initial DC priming become relevant.

In the response to *Salmonella*-2W1S, deletion of OX40L using the Rorc^cre^ resulted in the loss of IFNγ-producing Th1 CD4 T cells in both the draining lymphoid tissue and the effector tissue (colon). While T cell provision of OX40L cannot be excluded, these data are consistent with ILC3 OX40L expression critical for intestinal T cell responses, significantly developing previous studies using immunodeficient mice^50,55^ where ILC frequencies are grossly perturbed^31^. The lack of a role for ILC3s in the splenic response to *Lm-*2W1S may reflect the acute response or simply perhaps the very low frequency of ILC3 in the spleen and the lack of clear clusters of ILC3, as seen in the intestine and their draining LNs^30^. Within the intestinal tract, it is evident that ILC3s restrict responses to commensal bacteria not only at the level of effector T cell responses^56,57^ but also through T follicular helper cells and IgA production^58^. Thus ILC3s promote some T cell responses, while limiting others, indicating a critical regulatory role in intestinal immunity, potentially presenting an attractive cellular target for therapeutic manipulation^59^. Exactly where ILC3s interact with activated T cells remains unanswered; however, the impaired 2W1S-specific response observed in both the mLN and colon suggests a role in the draining lymphoid tissue, and the interfollicular spaces of the mLN may be the critical microenvironment fostering such crosstalk^31^.

The 2W1S-specific CD4 T cell response to *Lm*-2W1S has been characterised in detail^38^ and contains both T-bet-dependent Th1 effector cells and Bcl-6-dependent follicular T cells in roughly equal proportions. How the individual cells within this polyclonal response are pushed down one differentiation pathway or another is poorly understood but at least partially reflects differences in the strength of TCR signalling^60^. Our data suggest that only about half the activated CD4 T cells responding to *Lm*-2W1S ever express OX40 and differentiate into Th1 effector cells through upregulation of Blimp-1 and T-bet, alongside repression of Bcl-6^35,61^. Within the chronic *Salmonella* model, deletion of OX40L on ILC3 and T cells again resulted in a significant loss in the number Th1 effector cells; however, those cells that survived still expressed IFNγ. Thus signals through OX40 support Th1 effector cell expansion and survival rather than CD4 T cell differentiation^9^. The central finding of this manuscript is that, depending on the nature of the response, provision of OX40L signals is achieved through quite distinct interactions, with critical contributions by DCs and ILC3 demonstrated in vivo.

Collectively our data reveal that, despite the ability of many cell types to express costimulatory ligands for T cells, in vivo, there are specific cellular interactions that are needed for provision of these signals. Understanding these regulatory checkpoints and how costimulatory molecule expression is controlled in these situations will present further opportunities to better manipulate T cell responses for therapeutic benefit.

## Methods

### Mice

All mice used were C57BL/6 background and bred at Birmingham, except for IL-12p35^−/−^ mice that were generated at the University of Manchester and shipped to Birmingham. Strains used were C57BL/6 (WT), CD11c^cre^, Cd1d1^−/−^ (JAX stock 017294), E8111^cre^^62^, Great × Smart17A^36^, H2-Ab1^f/f^^63^, IL-12p35^−/−^ (JAX stock #002692)^64^, IFNγ^−/−^, IFNγR^f/f^ (JAX stock #025394)^65^, mT/mG (JAX stock #007576)^66^, OX40^−/−^, OX40^−/−^ × CD30^−/−^^13^, OX40L^f/f^^39^, PGK^cre^^67^ and Rorc^cre^^68^. Animals were used in accordance with Home Office guidelines at the University of Birmingham. Mice were housed at 21 °C +/− 2 °C, 55% humidity (+/−10%) with 12-h light/dark cycle in 7–7 IVC caging with environmental enrichment of plastic houses plus paper bedding.

### *Lm*-2W1S infection

Mice were infected with 10^7^ *actA-*deficient *Lm-*expressing OVA-2W1S (*Lm*-2W1S, kind gift from Dr. M. Jenkins) through intravenous injection in the tail vein. Bacteria were pre-cultured in 10 ml of LB broth containing 20 μg/ml chloramphenicol from a single colony and grown overnight at 37 °C at 200–250 rpm. Next day, 1 ml of *Lm*-2W1S was seeded into 200 ml of LB broth containing 20 μg/ml chloramphenicol and grown at 37 °C at 200–250 rpm until OD600 of 0.6–0.7 was reached. The liquid bacterial cultures were then centrifuged for 20 min at 4 °C, 4000 rpm. The supernatant was discarded and pellets were resuspended in LB broth with 15% glycerol. Glycerol stocks of *Lm*-2W1S were aliquoted into 1 ml cryovials and stored at −80 °C. Three randomly chosen samples were used to assess the viability of bacterial stocks by plating serial dilution of bacteria on agar containing 20 μg/ml chloramphenicol. Prior administration, the 1 ml *Lm*-2W1S was centrifuged and washed with sterile phosphate-buffered saline (PBS) before resuspending in adequate volume of sterile PBS to obtain 10^7^ bacteria per 200 μl.

### *Salmonella*-2W1S infection

The mice were given 20 mg of streptomycin (Sigma-Aldrich) in 100 μl sterile PBS by oral gavage 24 h prior to the administration of *Salmonella enterica* serovar Typhimurium BRD509 strain expressing the 2W1S epitope^49^ (*Salmonella*-2W1S, kind gift from Dr. S. McSorley). Bacteria were resuspended in adequate volume of sterile PBS to obtain 10^9^ bacteria per 100 μl and administered by oral gavage.

Glycerol stocks were prepared using the same method as described in previous section. Streptomycin was used as a selection antibiotic. The viability of the stocks was tested by randomly choosing 3 samples and plating the bacteria on MacConkey agar (Sigma-Aldrich) containing 100 μg/ml of streptomycin (Sigma-Aldrich).

### In vivo Ab administration

The depletion of NK cells and CD8 T cells was achieved by intraperitoneal (i.p.) administration of a dose of 250 μg anti-NK1.1 and 400 μg anti-CD8 in PBS at 48 h prior infection with 10^7^ *Lm*-2W1S. Abs were provided by AstraZeneca.

### Cell culture

Cell suspensions were prepared in sterile conditions.

*Experiments to assess OX40L expression in conditional OX40L-deficient mice*: Assessment of OX40L expression on Ag-presenting ells, like dendritic cells and B cells, required overnight culture with CD40 stimulation. For this purpose, purified anti-CD40 Ab was added at 1 μg/ml. To assess the expression of OX40L on T cells, 2 × 10^6^ splenocytes were cultured at 37 °C, 5% CO_2_ for 72 h in the presence of 10 μg/ml Abatacept and 0.5 μg/ml functional-grade anti-CD3ε.

*Experiments to assess expression and regulation of OX40L on splenic DCs*: In all, 2 × 10^6^ splenocytes from uninfected or *Lm*-2W1S infected mice were cultured overnight (18–24 h) at 37 °C, 5% CO_2_ in 24-well plate in 1 ml culture media (RPMI/10% foetal bovine serum/L-Glu/penicillin and streptomycin), either alone or with 1 µg anti-CD40 Abs or 100 ng recombinant IFNγ (Peprotech).

*Experiments to detect IFNγ production by NK cells*: Splenocytes from mice infected with *Lm*-2W1S 24 h previously were cultured for 3 h in culture media in the presence of brefeldin A (10 µg/ml) and then analysed by flow cytometry.

### Flow cytometry

Spleens were teased using fine forceps and digested for 25 min in RPMI containing with 250 µg/ml Collagenase-Dispase (Roche) and 25 µg/ml DNase I (Sigma). Digested tissue was then crushed through a nylon mesh, treated with Gey’s solution red blood lysis buffer and resuspended in appropriate volume of staining buffer. Splenocytes were re-filtered prior to use. Staining for 2W1S:I-A^b^ MHCII Tetramer and CXCR5 was performed for 1 h at room temperature (RT). Surface staining was performed at +4 °C for 30 min. Intracellular staining was done using the FoxP3 Fixation and Permeabilisation Kit (eBioscience) or Cytofix/Cytoperm Plus (BD Biosciences), both according to the manufacturer’s instructions. Abs raised against the following mouse agents were used: B220 (1:300 or 1:200; clone RA3-6B2, eBioscience), CCR6 (1:100; clone 29-2L17, eBioscience), CD3 (1:100 or 1:200; clone 145-2C11 or 17A2; eBioscience, BD Biosciences, or BioLegend), CD4 (1:200 or 1:300; clone RM4^−^5, BioLegend, eBioscience), CD8 (1:100 or 1:200; clone 53–6.7, eBioscience), CD11b (1:100 or 1:200; clone M1/70, eBioscience), CD11c (1:200 or 1:300; clone N418, eBioscience), CD25 (1:200; clone PC61, BioLegend), CD44 (1:200; clone IM7, eBioscience), CD49b (1:100; clone DX5, eBioscience), CD86 (1:100 or 1:600; clone GL-1, BioLegend), CXCR5 (1:50; clone 2G8, BD Biosciences), EOMES (1:50; clone Dan11mag, eBioscience), IFNγ (1:200 or 1:300; clone XMG1.2, eBioscience or BioLegend), IFNγR (1:100; clone 2E2, eBioscience), IL-7Rα (1:100; clone A7R34, BioLegend), Ly6c (1:800; clone HK1.4, BioLegend), MHCII (1:500; clone M5/114.15.2, eBioscience), NK1.1 (1:100; clone PK136, BD Biosciences or eBioscience), NKp46 (1:100; clone 29A1.4, BioLegend), OX40 (1:25; clone OX86, eBioscience), OX40L (1:50; clone RM134L, BioLegend), T-bet (1:50; clone 4B10, eBioscience), and TCRβ (1:100; clone H57–597, Biolegend). Addition of Spherotech Accucount blank particles was done to calculate cell frequencies. Flow cytometry was performed on a Fortessa analyser using the FACSDiva 8.0.2 software (BD), with data subsequently analysed with the FlowJo software version 10 (Tree Star).

### Immunofluorescence detection of IFNγ expression in situ

Procedure has been performed as previously described^69^. Briefly, WT mice were infected with 10^4^ colony-forming units *Lm*OVA. Mice were given 250 μg brefeldin A by i.p. injection, 6 h before being sacrificed. After 24 h, mice were euthanised, and the spleens were removed, fixed in 4% PFA and cryopreserved in OCT. Serial sections (30 µm in thickness) of frozen spleens were stained overnight at +4 °C in a humidified chamber with the following Abs: anti-B220 PB (1:300; 103230, Biolegend), anti-CD169 Alexa647 (1:300; 142407, Biolegend), anti-CD8 BV510 (1:100; 100751, Biolegend), anti-IFNγ BV421 (1:200; 505829, Biolegend), anti-I-A/I-E biotin (1:200; 107603, Biolegend), or anti-NKp46 (1:200; AF2225, R&D). For secondary detection, sections were then washed and incubated with Streptavidin-Cy3 (1:200; 016-160-084, Jackson Immunoresearch) and anti-goat IgG A647 (1:500; ab150131; Abcam) for 3 h at RT. All sections were analysed by confocal microscopy. Quantification of the interaction between DCs and IFNγ-producing cells was performed manually. DCs were randomly selected in the IFNγ-rich area. For each DC, the number of IFNγ-producing CD8 T cells and the number of IFNγ-producing NK cells was recorded and averaged.

### Human subjects

All analyses of human data were carried out in compliance with the relevant ethical regulations. Healthy blood donors gave informed consent at DRK Dresden, Germany, and buffy coats were obtained as approved by Charité ethics committee (EA4/059/17).

### In vitro culture of human cDC2 and NK cells

Peripheral blood mononuclear cells were isolated from buffy coats by density gradient centrifugation (Ficoll Paque Plus, GE Healthcare). CD56^+^ and CD19^+^ cells were depleted (CD56, CD19 microbeads and LD-columns, Miltenyi Biotec), the remaining cells were stained with biotinylated anti-CD1c Ab (1:50; 130-110-535, Miltenyi Biotec) and CD1c^+^ cells were enriched (anti-Biotin microbeads and LS-columns, Miltenyi Biotec). cDC2 were sorted from the CD1c-enriched fraction as viable CD14^−^ CD1c^+^ cells (Supplementary Fig. 8a). Pure NK cells were sorted on a FACS Aria II (BD) from the CD56^+^/CD19^+^ fraction as viable CD14^−^ CD19^−^ CD3^−^ CD56^+^ CD57^−^ lymphocytes (shown in Supplementary Fig. 8b), using anti-CD14 (1:50; 301820, Biolegend), anti-CD19 (1:50; 302216, Biolegend), anti-CD3 (1:50, generated in house), anti-CD56 (1:200, 318306, Biolegend) and anti-CD57 (1:50, 393304, Biolegend) Abs. Autologous NK cells and cDC2 were mixed at a ratio of 2:1 and cultured in RPMI-1640 (Gibco) supplemented with 10% foetal calf serum at the indicated conditions for 40 h. Where indicated, recombinant IFNγ (Miltenyi Biotec) was added at a concentration of 100 ng/ml and IL-12 and IL-15 (both Miltenyi Biotec) at 50 ng/ml each. After 40 h, cells were stained and analysed on a FACS Aria II (BD) using these additional Abs: anti-HLA-DR (1:200; generated in house), anti-CD86 (1:50; 130-114-095, Miltenyi), anti-OX40L (1:100; 326308, Biolegend), anti-CD40 (1:50; 334336, Biolegend), and anti-CD3 (1:50; 47-0036-42, eBioscience).

### RNA extraction of *Lm*-2W1S-stimulated DC and reverse transcriptase (RT) quantitative PCR

The DC [lin^−^ (B220, CD3, NK1.1), CD11c^+^, MHCII^+^, Ly6c^−^] were sorted at 4 h post infection with Lm-2W1S. Total RNA was extracted from Buffer RLT with 1% β-mercaptoethanol (Sigma-Aldrich)-conserved DC using the RNeasy® Mini Kit (Qiagen), according to the manufacturer’s instructions. RNA samples were stored at −80 °C until conversion into cDNA.

RNA was reverse-transcribed with SuperScript^TM^ III RT (Invitrogen^TM^ ThermoFisher Scientific) using oligo-dT strategy and according to the manufacturer’s instructions. To synthesise cDNA, 1 μl of oligo (dT) primer, 1 μl of 10 mM stock of dNTPs (Invitrogen) and 11 μl of total RNA in Nuclease-free water were incubated at 65 °C for 5 min and then put on ice for 3 min. The following reagents were added: 4 μl of 5× First strand buffer (Invitrogen), 1 μl RNase OUT (Invitrogen), 1 μl of 0.1 M dithiothreitol (Invitrogen) and 1 μl of 200 units/μl Superscript III RT (Invitrogen) followed by incubation at 50 °C for 60 min. Reaction was inactivated for 15 min at 72 °C. To remove RNA complementary to the cDNA, 1 μl (2 units) of RNase H were added, and the reaction mixture was incubated at 37 °C for 20 min. cDNA samples were stored as multiple aliquots at −20 °C for subsequent use.

### Quantitative real-time PCR

cDNA was amplified with the SensiFAST^TM^ SYBR® Hi-Rox Kit (Bioline Meridian Bioscience), according to the manufacturer’s instructions. Amplification reactions and detections were performed on ABI PRISM 7900HT Sequence Detection System (Applied Biosystems^TM^ ThermoFisher Scientific): 50 °C 2 min, 95 °C 10 min, [95 °C 15 s; 60 °C 1 min]  ×  40.

Primers used were Il12a p35 (Mm_Il12a_1_SG Qiagen QT01048334) and Il12b p40 (Mm_Il12b_1_SG Qiagen QT00153643). Each sample was run in triplicates, and the levels of the mRNA detected were normalised to β-actin. Data were analysed by calculating the relative expression of the target to β-actin in each sample = 2^ΔCt^ (with ΔCt = Ct_β-actin_ − Ct_target_).

### Statistical analysis

Data were analysed using GraphPad Prism (version 8). Non-parametric Mann–Whitney, two tailed or non-parametric Kruskal–Wallis one-way analysis of variance (ANOVA) with post hoc Dunn’s tests or, following the successful normality tests, ordinary one-way ANOVA with post hoc Tukey’s tests were used to determine significance, which was set at *p* ≤ 0.05. Median values were calculated and used in all analyses unless stated.

### Reporting summary

Further information on research design is available in the Nature Research Reporting Summary linked to this article.

## Supplementary information

Supplementary Information

Reporting Summary

## Data Availability

All data are available on request. Source data are provided with this paper.

## Source data

Source Data
