# Supplementary material for: Th1 responses in vivo require cell-specific provision of OX40L dictated by environmental cues
Source: Nat Commun. 2020 Jul 9;11:3421. doi: 10.1038/s41467-020-17293-3 (PMC7347572; doi:10.1038/s41467-020-17293-3)
Supplement: Supplementary file 1 — Supplementary Information [file 41467_2020_17293_MOESM1_ESM.pdf]

Th1 responses *in vivo* require cell-specific provision of OX40L dictated by environmental cues

Gajdasik et al., 2020

Supplementary Figures

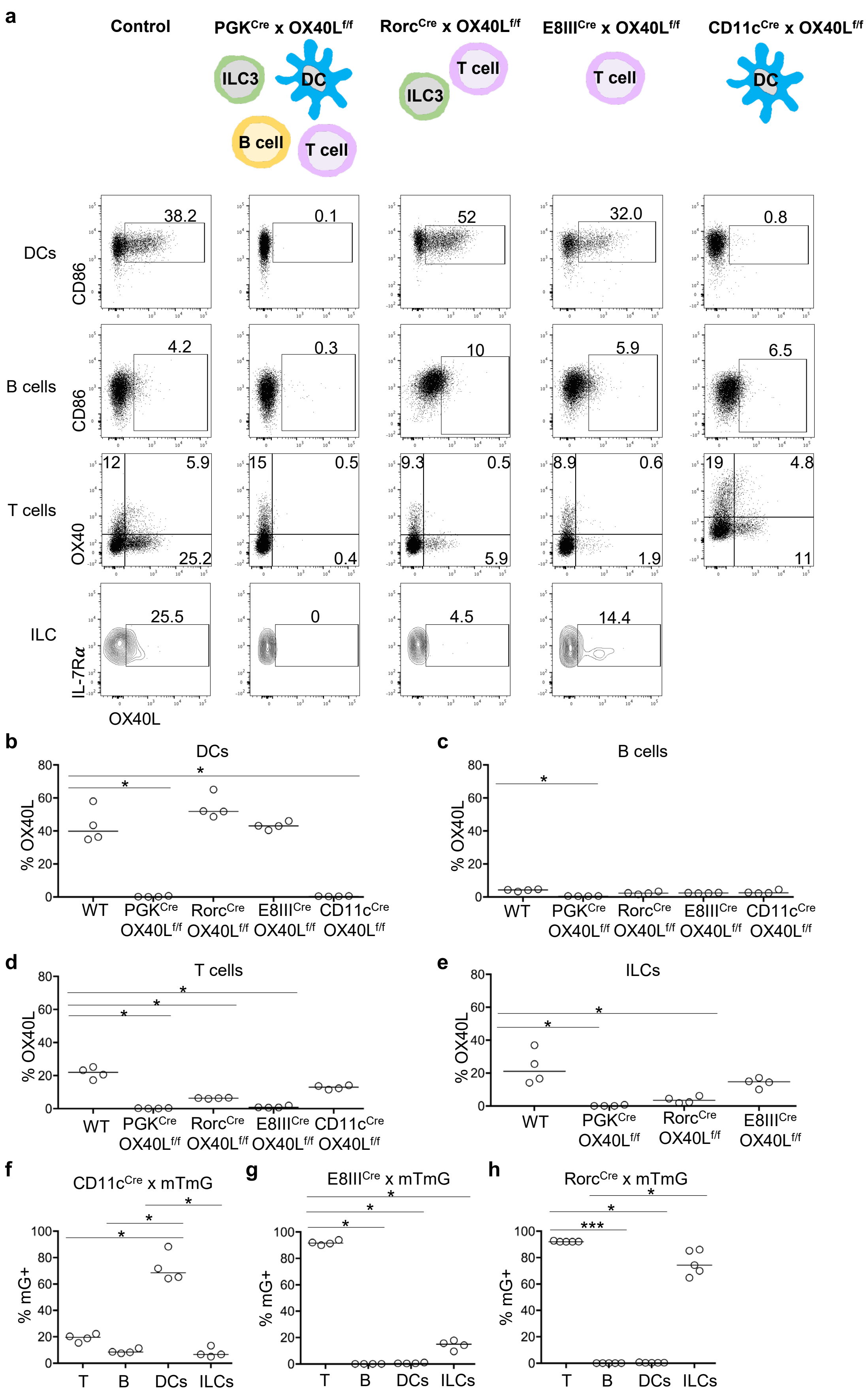
a
Control
PGKCre x OX40Lf/f
RorcCre x OX40Lf/f
E8IIICre x OX40Lf/f
CD11cCre x OX40Lf/f
ILC3
DC
B cell
T cell
DCs
B cells
T cells
ILC
CD86
OX40
IL-7Rα
OX40L
38.2
0.1
52
32.0
0.8
4.2
0.3
10
5.9
6.5
12
5.9
25.2
15
0.5
0.4
9.3
0.5
5.9
8.9
0.6
1.9
19
4.8
11
25.5
0
4.5
14.4
b
DCs
c
B cells
d
T cells
e
ILCs
% OX40L
WT
PGKCre OX40Lf/f
RorcCre OX40Lf/f
E8IIICre OX40Lf/f
CD11cCre OX40Lf/f
f
CD11cCre x mTmG
g
E8IIICre x mTmG
h
RorcCre x mTmG
% mG+
T
B
DCs
ILCs
*
***

Supplementary Figure 1. Analysis of OX40L expression in conditional OX40L-deficient mice.

To confirm appropriate deletion of OX40L in novel OX40L conditional mouse strains, splenocytes were cultured overnight and OX40L expression of key cell types assessed. **a** Expression of OX40L by DCs ($CD11c^+$, $MCHII^+$), B cells ($B220^+$) , T cells ($CD3^+CD4^+$) and ILCs (lin^- (CD3, B220, CD11c, CD11b), IL-7Rα^+, $CD4^+$,$CCR6^+$) in control versus $PGK^{cre} \times OX40L^{f/f}$, $Rorc^{cre} \times OX40L^{f/f}$, $E8111^{cre} \times OX40L^{f/f}$, and $CD11c^{cre} \times OX40L^{f/f}$ mice. **b** Expression of OX40L by DC in WT mice versus conditional OX40L deficient mice. **c** Expression of OX40L by B cells in WT mice versus conditional OX40L deficient mice. **d** Expression of OX40L by T cells in WT mice versus conditional OX40L deficient mice. **e** Expression of OX40L by ILC3 in WT mice versus conditional OX40L deficient mice. Data representative of 2 independent experiments (n=4 mice per group). The fidelity of cre expression in the cre-expressing mouse strains used to selectively mG (membrane tagged GFP) is induced by cre expression. **f** The percentage of mG^+ T cells, B cells, DCs and ILCs in the spleen of $CD11c^{cre}$ x mT/mG mice. **g** The percentage of mG^+ T cells, B cells, DCs and ILCs in the spleen of $E8111^{cre}$ x mT/mG mice. **h** The percentage of mG^+ T cells, B cells, DCs and ILCs in the spleen of $Rorc^{cre}$ x mT/mG mice. Data pooled from 2 independent experiments (n=4 $CD11c^{cre} \times$ mT/mG mice, $E8111^{cre} \times$ mT/mG mice, n=5 for $Rorc^{cre} \times$ mT/mG mice). Values on flow cytometry plots represent percentages, bars on scatter plots represents the median. Statistical significance was tested using an unpaired, non-parametric, Mann-Whitney two tailed T test: $*p \le 0.05$, $**p \le 0.01$, $***p \le 0.001$, $****p \le 0.0001$.

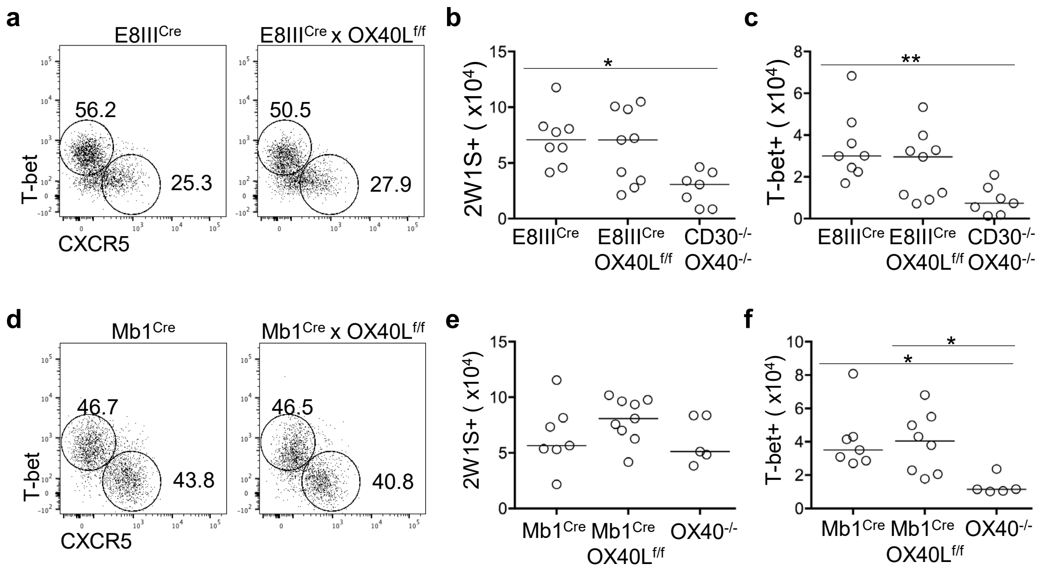

Supplementary Figure 2. Expression of OX40L by neither T cells nor B cells is required for generation of Th1 effector T cells in response to Lm-2W1S.
To investigate whether lymphocytes were key cellular sources of OX40L *in vivo*, the response to Lm-2W1S in T cell (E8111^{cre}) and B cell (Mb1^{cre}) conditional OX40L deficient mice was assessed. **a** Expression of T-bet versus CXCR5 by 2W1S-specific CD44^{hi} CD4 T cells in E8111^{cre} and E8111^{cre} × OX40L$^{f/f}$ mice. **b** Enumeration of CD44^{hi} 2W1S-specific CD4 T cells in E8111^{cre}, E8111^{cre} × OX40L$^{f/f}$ and CD30$^{-/-}$ x OX40$^{-/-}$ control mice. **c** Enumeration of T-bet^{+} 2W1S-specific CD44^{hi} CD4 T cells in E8111^{cre}, E8111^{cre} × OX40L$^{f/f}$ and CD30$^{-/-}$ × OX40$^{-/-}$ mice. Data representative of 2 independent experiments (n=8 E8111^{cre} mice, n =9 E8111^{cre} × OX40L$^{f/f}$ mice, n=7 CD30$^{-/-}$ × OX40$^{-/-}$ mice). **d** Expression of T-bet versus CXCR5 by 2W1S-specific CD44^{hi} CD4 T cells in Mb1^{cre} and Mb1^{cre} × OX40L$^{f/f}$ mice. **e** Enumeration of CD44^{hi} 2W1S-specific CD4 T cells in Mb1^{cre}, Mb1^{cre} × OX40L$^{f/f}$ and OX40$^{-/-}$ mice. **f** Enumeration of T-bet^{+} 2W1S-specific CD44^{hi} CD4 T cells in Mb1^{cre}, Mb1^{cre} × OX40L$^{f/f}$ and OX40$^{-/-}$ mice. Data pooled from 2 independent experiments (n=7 Mb1^{cre} mice, n=9 Mb1^{cre} × OX40L$^{f/f}$ mice, n=5 OX40$^{-/-}$ mice). Values on flow cytometry plots represent percentages, bars on scatter plots represents the median. Statistical significance was tested using Kruskal-Wallis one-way ANOVA with post hoc Dunn's test: *p≤0.05, **p≤0.01, ***p≤0.001, ****p≤0.0001.

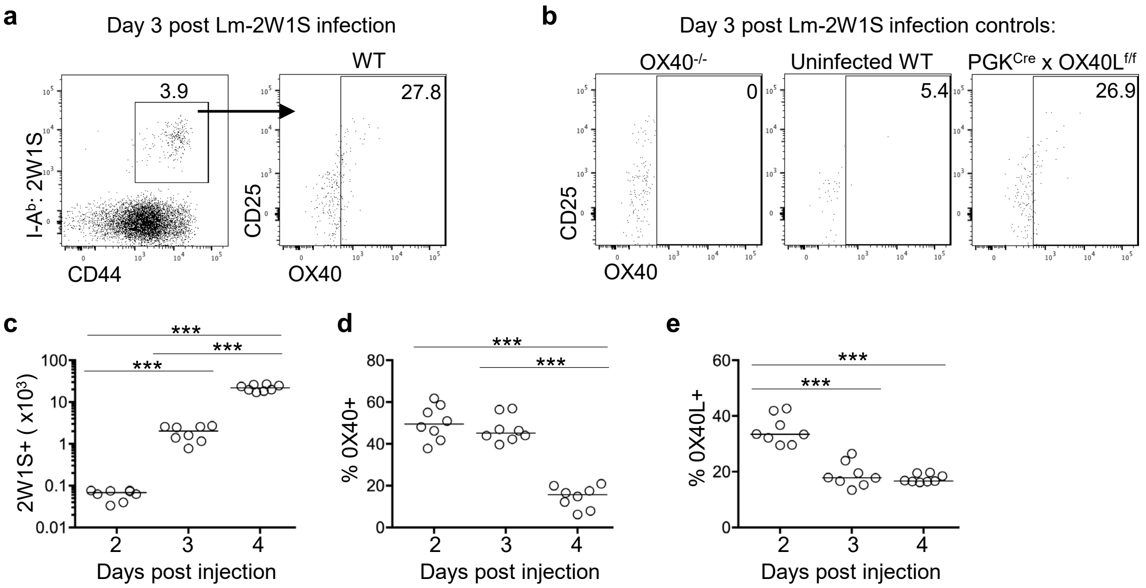

Supplementary Figure 3. Kinetics of OX40 and OX40L expression in response to Lm-2W1S.
To understand the relationship between DC OX40L expression and CD4 T cell OX40 expression, mice were infected with Lm-2W1S and T cells and DC from the same spleen analysed by flow cytometry. Expression of OX40 on 2W1S- specific CD4 T cells at day 3 post infection with Lm-2W1S in (**a**) WT and (**b**) uninfected, OX40$^{-/-}$ and PGK^{Cre} x OX40L$^{f/f}$ control mice. **c** Enumeration of 2W1S- specific CD4 T cells at day 2, 3 and 4 post infection with Lm-2W1S. **d** Percentage of 2W1S- specific CD4 T cells expressing OX40 at 2, 3 and 4 days post infection with Lm-2W1S. **e** Percentage of DC expressing OX40L at day 2, 3 and 4 post infection with Lm-2W1S. Data pooled from 2 independent experiments (n=8 mice per group). Values on flow cytometry plots represent percentages, bars on scatter plots represents the median. Statistical significance was tested using an unpaired, non-parametric, Mann-Whitney two tailed T test: *p≤0.05, **p≤0.01, ***p≤0.001, ****p≤0.0001.

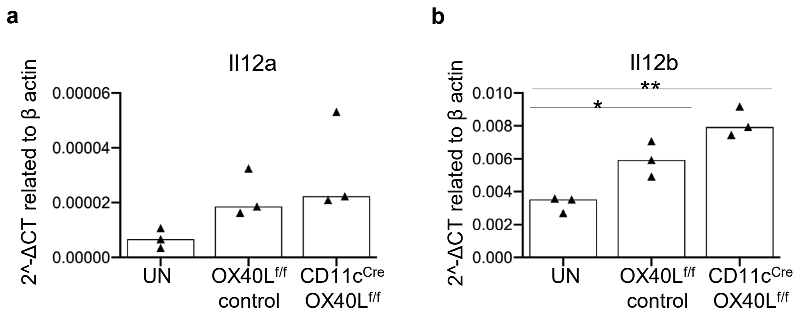

Supplementary Figure 4. Optimal levels of IL-12 in CD11c^{Cre} x OX40L$^{f/f}$.
To determine the levels of IL-12 (p40) and IL-12 (p35) mRNA in CD11c^{Cre} x OX40L$^{f/f}$ versus OX40L$^{f/f}$ mice, splenic DCs were sorted at 4hrs post infection with Lm-2W1S and their total RNA extracted, reverse transcribed, and amplified by real-time PCR using primers specific for IL-12(p40), IL-12(p35), or β-actin. **a** Levels of IL-12a quantified and normalized against β-actin. **b** Levels of IL-12b quantified and normalized against β-actin. Data from 1 experiment (n= 3 mice per group). Statistical significance was tested in using ordinary one-way ANOVA with post hoc Tukey's tests, following successful normality tests: *p≤0.05, **p≤0.01, ***p≤0.001, ****p≤0.0001.

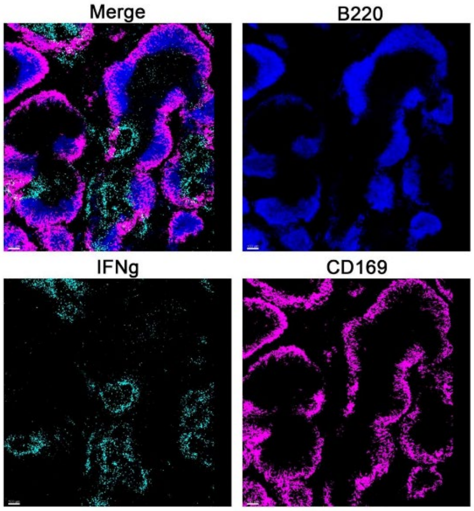

Supplementary Figure 5. Localisation of IFNγ-expressing cells in the spleen post infection with *Listeria monocytogenes*.

To identify where cells producing IFNγ were located within the spleen, WT mice were infected with *Listeria monocytogenes*, and killed 24 h after infection, with treatment with BFA conducted 6 hrs before spleen harvest. Spleen sections were stained with B220 (B cells, blue), CD169 (Marginal zone Macrophages, magenta), and IFNγ (cyan) antibodies. Photograph is representative of IFNγ localisation in the spleen, scale bar: 100 μm. Data representative of 2 independent experiments (n=2).

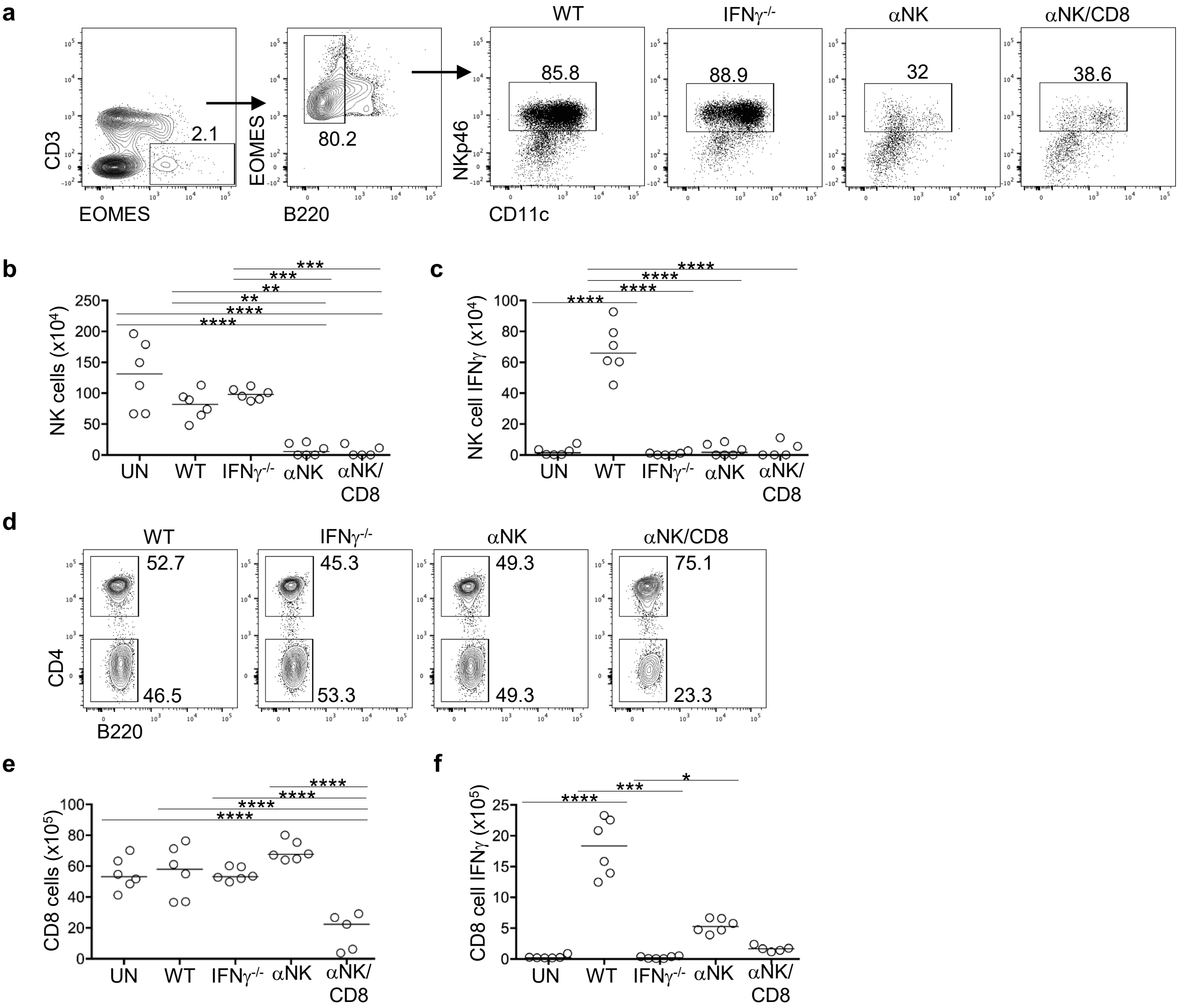

Supplementary Figure 6. *In vivo* depletion of NK cells and CD8 T cells.

To confirm appropriate depletion of NK cells and CD8 T cells and the resulting expression of early IFNγ responses, the splenocytes were cultured ex vivo for 3 hrs in the presence of BFA. **a** Gating strategy used to identify NK cells in WT, IFNγ$^{-/-}$, and αNK and αNK/CD8 (pre-treated with αNK and αNK and αCD8 monoclonal antibodies respectively) mice. **b** Total numbers of NK cells in uninfected (UN), WT, IFNγ$^{-/-}$, and αNK and αNK/CD8 mice at 24 hrs post infection with Lm-2W1S. **c** Total numbers of IFNγ producing NK cells in UN, WT, IFNγ$^{-/-}$, and αNK and αNK/CD8 mice at 24 hrs post infection with Lm-2W1S. **d** Assessment of CD8 T cells depletion as shown by the comparison of the CD3$^+$CD4$^+$ and CD3$^+$CD4$^-$ populations. **e** Total numbers of CD8 T cells identified in UN, WT, IFNγ$^{-/-}$, and αNK and αNK/CD8 mice. **f** Total numbers of IFNγ producing CD8 T in UN, WT, IFNγ$^{-/-}$, and αNK and αNK/CD8 mice. Data pooled from 2 independent experiments (n=6 uninfected WT mice, n=6 WT mice, n=6 IFNγ$^{-/-}$ mice, n=6 αNK treated mice, n=5 αNK/CD8 treated mice. Values on flow cytometry plots represent percentages, bars on scatter plots represents the median. Statistical significance was tested using ordinary One-way ANOVA with post hoc Tukey's tests, following normality tests: *p≤0.05, **p≤0.01, ***p≤0.001, ****p≤0.0001.

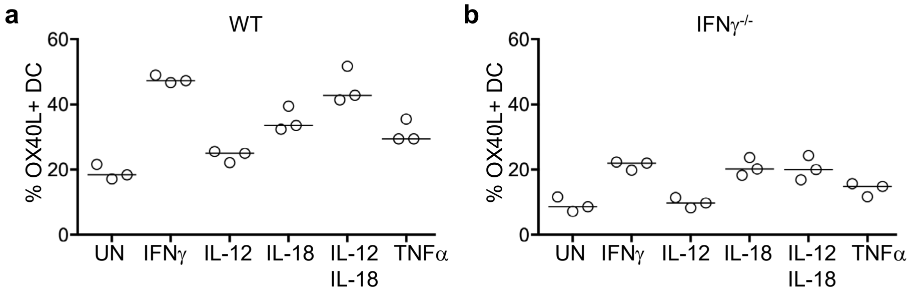

Supplementary Figure 7. IL-18 stimulates OX40L expression by DC.
To investigate other potential signals that might affect OX40L expression by splenic DC, splenocytes isolated from WT and IFN$\gamma^{-/-}$ mice were cultured *in vitro* with recombinant IFNγ, IL-12, IL-18, IL-12 and IL-18 and TNFα. Proportion of splenic DC from WT **(a)** and IFN$\gamma^{-/-}$ (**b**) mice expressing OX40L post overnight culture. Data representative of 2 independent experiments (n=3 mice per group).

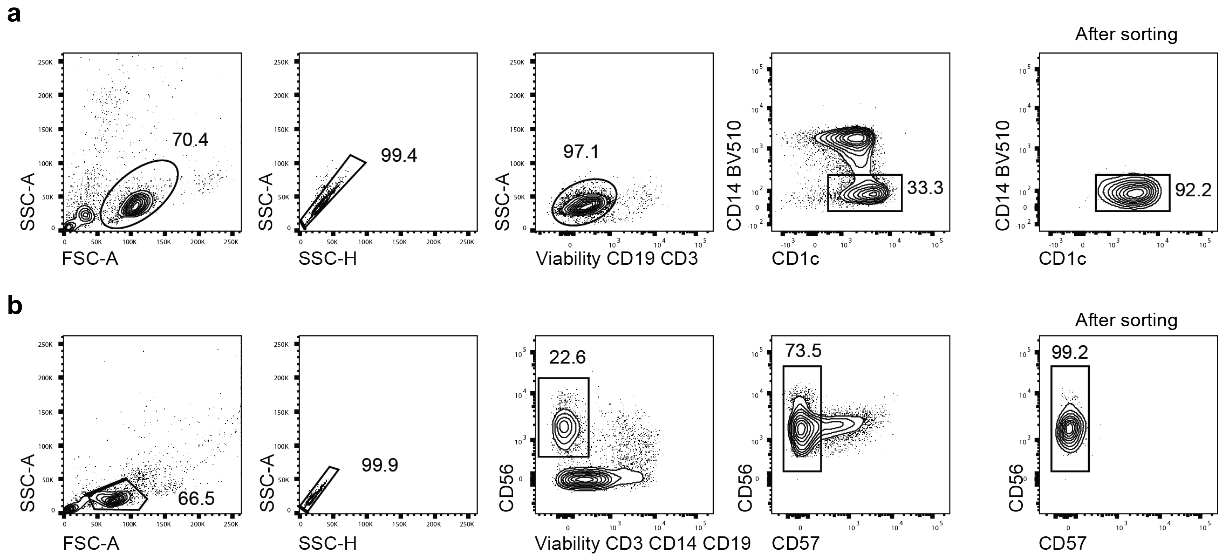

Supplementary Figure 8. Gating strategy for isolation of human cDC2 and NK cells by FACS.

Sorting strategies for co-cultures of human NK cells and cDC2. **a** cDC2 were sorted as viable $CD3^-$ $CD14^-$ $CD19^-$ $CD1c^+$ cells. **b** NK cells were sorted as viable $CD3^-$ $CD14^-$ $CD19^-$ $CD56^+$ $CD57^-$ cells.
