# Supplementary material for: Th1 responses in vivo require cell-specific provision of OX40L dictated by environmental cues
Source: Nat Commun. 2020 Jul 9;11:3421. doi: 10.1038/s41467-020-17293-3 (PMC7347572; doi:10.1038/s41467-020-17293-3)
Supplement: Supplementary file 2 — Reporting Summary [file 41467_2020_17293_MOESM2_ESM.pdf]

nature research

Corresponding author(s): David Withers

Last updated by author(s): Jun 17, 2020

Reporting Summary

Nature Research wishes to improve the reproducibility of the work that we publish. This form provides structure for consistency and transparency in reporting. For further information on Nature Research policies, see Authors & Referees and the Editorial Policy Checklist .

Statistics

For all statistical analyses, confirm that the following items are present in the figure legend, table legend, main text, or Methods section.

n/a	Confirmed	
☐	☒	The exact sample size (*n*) for each experimental group/condition, given as a discrete number and unit of measurement
☒	☐	A statement on whether measurements were taken from distinct samples or whether the same sample was measured repeatedly
☐	☒	The statistical test(s) used AND whether they are one- or two-sided<br>*Only common tests should be described solely by name; describe more complex techniques in the Methods section.*
☒	☐	A description of all covariates tested
☐	☒	A description of any assumptions or corrections, such as tests of normality and adjustment for multiple comparisons
☐	☒	A full description of the statistical parameters including central tendency (e.g. means) or other basic estimates (e.g. regression coefficient) AND variation (e.g. standard deviation) or associated estimates of uncertainty (e.g. confidence intervals)
☐	☒	For null hypothesis testing, the test statistic (e.g. *F*, *t*, *r*) with confidence intervals, effect sizes, degrees of freedom and *P* value noted<br>*Give P values as exact values whenever suitable.*
☒	☐	For Bayesian analysis, information on the choice of priors and Markov chain Monte Carlo settings
☒	☐	For hierarchical and complex designs, identification of the appropriate level for tests and full reporting of outcomes
☒	☐	Estimates of effect sizes (e.g. Cohen's *d*, Pearson's *r*), indicating how they were calculated

Our web collection on statistics for biologists contains articles on many of the points above.

Software and code

Policy information about availability of computer code

Data collection	BC FACSDiva software (8.0.2) was used for collection of flow cytometric data; Applied Biosystems 7900HT Fast Real-Time PCR system software (SDS Plate Utility v2.2) was used for collection of qPCR data.
Data analysis	FlowJo (V10) was used for flow cytometric analysis. Prism (V8, by GraphPad) were used for the generation of graphs and statistical analysis.

For manuscripts utilizing custom algorithms or software that are central to the research but not yet described in published literature, software must be made available to editors/reviewers. We strongly encourage code deposition in a community repository (e.g. GitHub). See the Nature Research guidelines for submitting code & software for further information.

Data

Policy information about availability of data

All manuscripts must include a data availability statement. This statement should provide the following information, where applicable:

- Accession codes, unique identifiers, or web links for publicly available datasets
- A list of figures that have associated raw data
- A description of any restrictions on data availability

All data available on request.

Field-specific reporting

Please select the one below that is the best fit for your research. If you are not sure, read the appropriate sections before making your selection.

☒ Life sciences ☐ Behavioural & social sciences ☐ Ecological, evolutionary & environmental sciences

October 2018

Life sciences study design

All studies must disclose on these points even when the disclosure is negative.

Sample size	Initial power calculations were performed for 2W1S-specific T cell populations in WT versus genetically modified mice, which proposed group sizes of at least 9 mice per group. Given the reproducibility of the endogenous 2W1S-specific response, we sought to reach this group size by pooling samples from independent experimental repeats, with the aim of of repeating all in vivo experiments three times, but at least twice, dependent on mouse availability. Group sizes greater than 9 resulted from an abundance of mice of that genotype. For some complex crosses we were unable to generate group sizes of 9. Group sizes for CD1d-/- and Il12-/- mice were dictated by the number of available mice provided by collaborators. Separate power calculations were used to determine group sizes for OX40L expression experiments. For all other experiments, power calculations were not performed, but a principle of an 'n' of 5-6 was sought from atleast two independent experiments, dependent upon sample availability.
Data exclusions	None
Replication	All in vivo experiments were performed at least twice, in many cases three times and the number of experiments is described throughout. All attempts at replication were successful. The 2W1S response is highly reproducible in C57BL/6 mice enabling pooling of data sets which was done where ever possible.
Randomization	Experimental groups were not randomized and frequently reflected the availability of sufficient mice of a given genotype, although experimental groups were age and sex matched where possible (and in most cases this was achieved).
Blinding	Investigators were not blinded for any experiments described in the study. For the vast majority of experiments, flow cytometry was used to determine cellular phenotypes and enumerate populations and as these data were not subjective, blinding was not considered necessary and the data generated was directly recorded and used. As described above, no data was excluded.

Reporting for specific materials, systems and methods

We require information from authors about some types of materials, experimental systems and methods used in many studies. Here, indicate whether each material, system or method listed is relevant to your study. If you are not sure if a list item applies to your research, read the appropriate section before selecting a response.

Materials & experimental systems

n/a	Involved in the study	
☐	☒	Antibodies
☒	☐	Eukaryotic cell lines
☒	☐	Palaeontology
☐	☒	Animals and other organisms
☐	☒	Human research participants
☒	☐	Clinical data

Methods

n/a	Involved in the study	
☒	☐	ChIP-seq
☐	☒	Flow cytometry
☒	☐	MRI-based neuroimaging

Antibodies

Antibodies used

The following anti-mouse antibodies were used for flow cytometry:
[antibody (Company, clone, catalogue number, conjugation, dilution)]
anti-B220 (Thermo Fisher Scientific, RA3-6B2, 11-0452-82 or 48-0452-82, FITC or eFluor450, 1:300 or 1:200)
anti-CCR6 (Biolegend, 29-2L17, 129819, BV605, 1:100)
anti-CD3ε (Thermo Fisher Scientific, BD Biosciences and Biolegend, 145-2C11 or 17A2, 56-0032-82, 553062, 100229 or 100215, Alexa Fluor 700, FITC or BV650, 1:100 or 1:200)
anti-CD4 (Biolegend or Thermo Fisher Scientific, RM4-5, 100553 or 17-0042-82, BV510 or APC, 1:300 or 1:200)
anti-CD8 (BioLegend or Thermo Fisher Scientific , 53-6.7, 100747 or 17-0081-82, BV711 or APC, 1:200 or 1:100)
anti-CD11b (Thermo Fisher Scientific, M1/70, 48-0112-82 or 11-0112-82, eFluor 450 or FITC, 1:200 or 1:100)
anti-CD11c (Thermo Fisher Scientific, N418, 25-0114-82, 48-0114-82 or 11-0114-85, PE-Cyanine7, eFluor 450 or FITC, 1:200 or 1:300)
anti-CD25 (Biolegend, PC61, 102037, BV650, 1:200),
anti-CD44 (BioLegend or Thermo Fisher Scientific, IM7, 103041 or 11-0441-82, BV785 or FITC, 1:200)
anti-CD49b (Thermo Fisher Scientific, DX5, 11-5971-82, FITC, 1:100)
anti-CD86 (Biolegend, GL-1, 105005 or 105035, FITC or BV650, 1:100 or 1:600)
anti-CXCR5 (BD Biosciences, 2G8, 560617, PE-Cyanine7, 1:50)
anti-EOMES (Thermo Fisher Scientific, 12-4875-82 or 25-4875-82, Dan11mag, PE or PE-Cyanine7, 1:50)
anti-IFNγ (Thermo Fisher Scientific and Biolegend, XMG1.2, 25-7311-82 and 505825, PE-Cyanine7, 1:200 or 1:300)
anti-IFNγR (Thermo Fisher Scientific, 13-1191-82, 2E2, Biotin, 1:100)

anti-IL-7Rα (Biolegend, A7R34, 135023, BV421, 1:100)
anti-Ly6c (Biolegend, HK1.4, 128037, BV711, 1:800)
anti-MHCII (BioLegend, M5/114.15.2, 107635, BV510, 1:500)
anti-NK1.1 (BD Bioscience or Thermo Fisher Scientific, PK136, 564143 or 11-5941-82, BV650 or FITC, 1:100)
anti-NKp46 (Biolegend, 29A1.4, 137619 or 137621, BV605 or BV711, 1:100)
anti-OX40 (Thermo Fisher Scientific, OX86, 17-1341-82 or 62-1341-82, APC or Super Bright 436, 1:25)
anti-OX40L (Biolegend, RM134L, 108806, PE, 1:50)
anti-T-bet (Thermo Fisher Scientific, 4B10, 12-5825-82 or 25-5825-82, PE or PE-Cyanine7, 1:50)
anti-TCRβ (Biolegend, H57-597, 109224, Alexa Fluor 700, 1:100)
The following antibodies were used for immunofluorescence:
[antibody (Company, clone, catalogue number)]
anti-B220 PB (Biolegend, clone RA3-6B2, 103230) 1:300
anti-CD169 Alexa647 (Biolegend, clone 3D6.112, 142407) 1:300
anti-CD8 BV510 (Biolegend, clone 53-6.7, 100751) 1:100
anti-IFNγ BV421 (Biolegend, clone XMG1.2, 505829) 1:200
anti-I-A/I-E biotin (Biolegend, clone M5/114.15.2, 107603) 1:200
anti-NKp46 (R&D, goat polyclonal, AF2225). 1:200
anti-goat IgG A647 (Abcam, donkey polyclonal, ab150131) 1:500
Streptavidin-Cy3 (Jackson Immunoresearch, 016-160-084) 1:200

The following anti-human antibodies were used for FACS:
[antibody (Company, clone, catalogue number)]
anti-CD1c (Miltenyi, REA694, 130-110-535) 1:50
anti-CD14 (Biolegend, M5E2, 301820) 1:50
anti-CD19 (Biolegend, HIB19, 302216) 1:50
anti-CD3 (Romagnani Lab, Ucht1) 1:50
anti-CD56 (Biolegend, HCD56, 318306) 1:200
anti-CD57 (Biolegend, QA17A04, 393304) 1:50
anti-HLA-DR (Romagnani Lab, 277) 1:200
anti-CD86 (Miltenyi, FM95, 130-114-095) 1:50
anti-OX40L (Biolegend, 11C3.1, 326308) 1:100
anti-CD40 (Biolegend, 5C3, 334336) 1:50
anti-CD3 (ebioscience, SK7, 47-0036-42) 1:50

Validation

All primary Abs were initially tested and titrated before routine use in the lab. Initial selection was based upon manufacturer's website and published data. Antibodies were validated using FMO controls, staining of genetically modified tissue and/or previously reported staining patterns.

Animals and other organisms

Policy information about studies involving animals; ARRIVE guidelines recommended for reporting animal research

Laboratory animals

All mice used were C57BL/6 background and bred at Birmingham, except for IL-12p35-/- mice which were generated at the University of Manchester by Professor Andrew MacDonald and shipped to Birmingham. Within experiments, mice were age and sex matched wherever possible, but both male and female mice were used experimentally. Mice were aged between 6 and 12 weeks of age at the start of the experiment. CD11ccre (JAX stock 008068), Cd1d1-/- (JAX stock 017294), mT/mG (JAX stock 007576), IFNγRf/f (JAX stock 025394) were obtained from The Jackson Laboratory. E8111cre mice were obtained from Dr. Alfred Singer. Great x Smart17A mice were obtained from Professor Richard Locksley. H2-Ab1f/f mice were obtained from Professor Daniela Finke. IFNγ-/- mice were obtained from Professor Adam Cunningham. OX40-/- and OX40-/- x CD30-/- mice were obtained from Professor Peter Lane. OX40Lf/f mice were obtained from Professor Marina Botto. Rorccre mice were obtained from Professor Dan Littman. Mb1cre mice were obtained from Professor Michael Reth.
Mice were housed at 21°C +/- 2°C, 55% humidity (+/- 10%) with 12 hr light dark/ cycle in 7-7 IVC caging with environmental enrichment of plastic houses plus paper bedding.

Wild animals

This study did not use wild animals

Field-collected samples

No field collected samples were used in the study

Ethics oversight

All animal experiments were conducted in accordance of Home Office Guidelines, authorized under PPL P33E83BE5 granted to D. Withers after ethical review by the Animal Welfare and Ethical Review Body at the University of Birmingham, UK

Note that full information on the approval of the study protocol must also be provided in the manuscript.

Human research participants

Policy information about studies involving human research participants

Population characteristics	Human PBMCs were isolated from deidentified buffy coats supplied by the German Red Cross (DRK-Blutspendedienst Nord-Ost). We do not have access to population characteristics apart from the general requirements for healthy blood donors (18-73 years of age, body weight of more than 50 kg). To explore whether human DC upregulate OX40L expression, samples from healthy donors under these conditions were considered sufficient.
Recruitment	Healthy blood donors gave informed consent at DRK Dresden, Germany and buffy-coats were randomly selected by the German Red Cross
Ethics oversight	Charité ethics committee (EA4/059/17)

Note that full information on the approval of the study protocol must also be provided in the manuscript.

Flow Cytometry

Plots

Confirm that:

- [x] The axis labels state the marker and fluorochrome used (e.g. CD4-FITC).
- [x] The axis scales are clearly visible. Include numbers along axes only for bottom left plot of group (a 'group' is an analysis of identical markers).
- [x] All plots are contour plots with outliers or pseudocolor plots.
- [x] A numerical value for number of cells or percentage (with statistics) is provided.

Methodology

Sample preparation	Tissue (spleen) was harvested and a single cell suspension created through physical disruption. RBCs were lysed and cells were stained, with antibodies
Instrument	Data collection was performed using a BD LSRFortessa X20 or FACS Aria II
Software	BD FACS DIVA (8.0.2) was used for data collection and FlowJo Software (v10) used for data analysis
Cell population abundance	Human cDC2 and NK cell populations were isolated by FACS to a purity of at least 92%. This was assessed by reanalysis of sorted cells.
Gating strategy	Gating strategies are described in detail in the manuscript for analysis experiments. The gating strategy for sorting populations of human cells is included in the Supplementary information

- [x] Tick this box to confirm that a figure exemplifying the gating strategy is provided in the Supplementary Information.
